# BAFF augments IgA2 and IL‐10 production by TLR7/8 stimulated total peripheral blood B cells

**DOI:** 10.1002/eji.201646861

**Published:** 2017-10-11

**Authors:** Gerco den Hartog, Thijs L.J. van Osch, Martijn Vos, Ben Meijer, Huub F.J. Savelkoul, R.J. Joost van Neerven, Sylvia Brugman

**Affiliations:** ^1^ Animal Sciences Group Cell Biology and Immunology group Wageningen University the Netherlands; ^2^ FrieslandCampina Amersfoort the Netherlands; ^3^ Centre for Immunology of Infectious Diseases National Institute for Public Health and the Environment Bilthoven the Netherlands

**Keywords:** CD38, Class switch recombination, CpG‐ODN, Plasma cell, Retinoic acid

## Abstract

Class‐switching of B cells to IgA can be induced via both T‐cell‐dependent and T‐cell‐independent mechanisms. IgA is most predominantly produced mucosally and is important for combating infections and allergies. In contrast to mice, humans have two forms of IgA; IgA1 and IgA2 with diverse tissue distribution. In early life, IgA levels might be sub‐optimal especially during the fall season when bacterial and viral infections are more common. Therefore, we investigated using human B cells whether T‐cell‐independent factors ‐promoting cell survival, class switching and immunoglobulin secretion‐ BAFF, APRIL, IL‐10 and retinoic acid can boost IgA production in the context of viral or bacterial infection. To this end total and naive peripheral blood B cells were stimulated with these factors for 6 days in the presence or absence of TLR7/8 agonist R848 (mimicking viral infection) or TLR9 agonist CpG‐ODN (mimicking bacterial infection). We show that BAFF significantly augments IgA2 production in TLR7/8 stimulated mature, but not naïve B cells. In addition, BAFF augments IL‐10 production and viability in TLR7/8 and TLR9 stimulated mature B cells. These data warrant further investigation of its role in immune regulation both in the periphery and mucosal tissues in early life or during disease.

## Introduction

In early life, at the time the immune system develops, exposure to environmental pathogens, such as viruses and bacteria can lead to severe illness. For example respiratory syncytial virus (RSV) infection and allergic wheezing might predispose children to develop asthma later in life 1, 2. Immune exclusion of these pathogens by boosting IgA might prevent these diseases by blocking initial infection. Understanding how to boost natural IgA responses might therefore help to prevent respiratory and gastrointestinal disease.

The outcome of an immune response is partly dependent on the molecules in the immediate environment of cells; the niche. Proper discrimination must take place between pathogenic and commensal bacteria as well as between food ingredients, toxic substances or viral particles. To this end a complex system of innate and adaptive immune cells bearing different pattern recognition receptors integrate all the different signals into the correct immune response.

One of the most important cells at the mucosal surface is the IgA‐producing plasma cell. To our current understanding T cell independent (TI) induction of IgA largely depends on innate tissue factors derived from epithelial and/or dendritic cells 3. The TNF‐α family members ‘a proliferation inducing ligand’ (APRIL) and ‘B cell activating factor’ (BAFF) produced by intestinal and respiratory tract epithelial cells and DCs can substitute for CD40 ligation in TI induction of IgA 4, 5, 6, 7. In healthy tissues TGF‐ß and IL‐10 are abundantly produced. Retinoic acid (RA) that is synthesized from retinal from dietary vitamin A by epithelial cells and dendritic cells, can be found in the intestinal tract and is important for gut tropism 8, 9, 10. Together, these factors can contribute to induction of IgA class switch recombination (CSR) and differentiation of B cells into plasma cells, probably regardless of the presence of T cells 4, 11. In serum BAFF and APRIL are critical factors involved in maintenance of the B cell pool and humoral immunity 12. Furthermore, during activation homing markers such as α4β7 (gut homing) or CCR10^+^ (skin, airway or gut homing) can also be induced to ensure proper homing of the immune cell to the exact tissue it first encountered the antigen and relevant secondary tissues 13, 14, 15.

In contrast to rodents, humans produce two subclasses of IgA (IgA1 and IgA2) that are present in different concentrations and ratios throughout the body 4, 16, 17, suggesting different pathways to regulate their production 18. In serum, the levels of IgA1 are higher than IgA2, whereas in the distal intestine IgA2 levels are higher than IgA1 19. Compared to IgA1, IgA2 has a shorter hinge region resulting in better resistance to bacterial proteolysis 18, 20. Of the two IgA subclasses, especially IgA2 seems to be associated with protection against inhalant allergens. For example, immunotherapy against grass pollen allergy clearly induced an allergen‐specific IgA2 antibody response that was associated with mucosal TGF‐β expression and reduced seasonal symptoms 21. Furthermore, we recently showed that house dust mite (HDM) allergic patients who suffered from rhinitis and eczema have significantly lower IgA2 levels compared to patients that suffer from rhinitis only 22. Apart from their important role as antibody producing cells, in the last decades it has been shown that B cells can also produce anti‐inflammatory cytokines such as IL‐10 that can actively suppress immune responses 23.

In this study, we set out to investigate whether IgA subclass production and B cell maturation are modulated by the T cell‐independent B cell class switch, survival and immunoglobulin inducing factors BAFF, APRIL, IL‐10 and RA in the context of bacterial or viral infection. Here we show that BAFF specifically enhances IgA2 production in TLR7/8 stimulated mature, but not naïve B cells from peripheral blood. In addition, BAFF augments IL‐10 production in TLR7/8 and TLR9 stimulated mature peripheral blood B cells.

## Results

### BAFF and RA differentially affect viability of stimulated mature B cells

**Figure 1:**
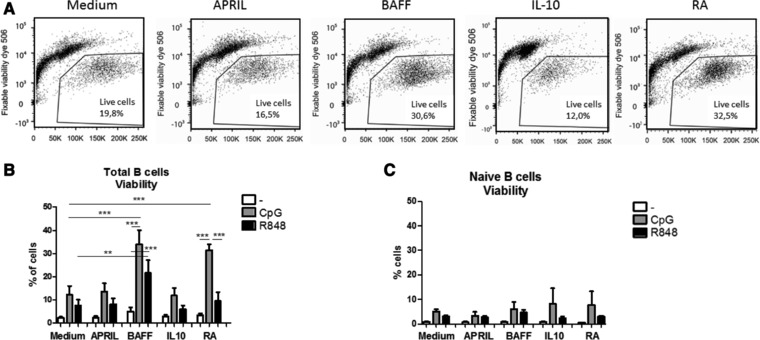
BAFF and retinoic acid differentially affect viability of stimulated mature B cells, (A) Representative flow cytometry plots for viability staining (CpG‐ODN), (B) Percentages of viable B cells (6 days of culture of total peripheral blood B cells) after stimulation with CpG‐ODN or R848 in combination with T cell independent B cell class switch inducing factors (mean +SEM, *n* = 5 donors per group, combined graph of three separate experiments, Repeated measures ANOVA, Tukey post‐hoc test, *p<0.05, **p<0.01, ***p<0.001), C) Percentages of viable B cells (6 days of culture of naive B cells) after stimulation with CpG‐ODN or R848 in combination with T cell independent B cell class switch inducing factors. Data shown as mean +SEM, *n* = 3–5 donors per group, combined graph of two separate experiments. *p<0.05, **p<0.01, ***p<0.001 Unpaired *t*‐test.

Since viability is crucial for effective immune responses, we assessed viability of the isolated B cells (>98% purity) after six days of culture. Unstimulated total or naive peripheral blood‐derived B cells without the presence of any other cell type show a very low viability after 6 days of culture (Fig. 1B and C). Stimulation with either CpG‐ODN (TLR9) or R848 (TLR7/8) increased the viability of total peripheral blood B cells in almost all conditions (Fig. 1A and B). BAFF and RA significantly enhanced the viability induced by CpG‐ODN, while BAFF also augmented the increased viability induced by R848. Addition of APRIL or IL‐10 to either CpG‐ODN or R848 did not change the viability compared to the medium controls. The viability did not exceed 5% after 6 days of culture when only naïve cells are cultured even in the presence of CpG‐ODN or R848 (Fig. 1C). Since BAFF has been reported to be a survival factor for B cells, we investigated whether memory and naive B cells have the appropriate receptors. While both naive (CD27‐) and memory (CD27^+^) B cells have BAFF‐R (receptor for BAFF), and TACI (receptor for APRIL and BAFF), we hardly observed IL‐10 receptor and BCMA (receptor for BAFF and APRIL) on naive and memory B cells at baseline. (Supporting Information Fig. 1). After 6 days of culture the percentages of TACI and IL‐10R remained the same, however in cells exposed to BAFF, a clear reduction in percentage of BAFF‐R+ B cells was observed (Supporting Information Fig. 1B). Addition of other cells (B cell depleted PMBCs) at the start of the culture enhanced the viability of the B cells greatly (Supporting Information Fig. 2).

### Retinoic acid induces CD38 expression in stimulated naïve and total peripheral blood B cells

**Figure 2:**
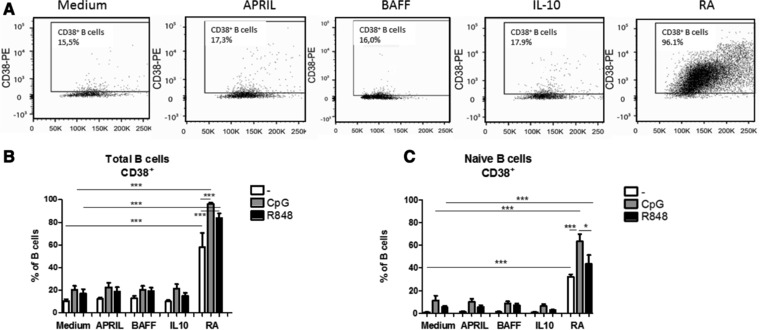
Retinoic acid increases the percentage of CD38 on naive and mature B cells, (A) Representative flow cytometry plots for CD38 staining, (B) Percentage of CD38^+^ B cells after 6 days of culture of total peripheral blood B cells after stimulation with CpG‐ODN or R848 in combination with T cell independent B cell class switch inducing factors (mean +SEM, *n* = 5 donors per group, combined graph of three separate experiments, Repeated measures ANOVA, Tukey post‐hoc test, *p<0.05, **p<0.01, ***p<0.001 (C) Percentage of CD38^+^ B cells after 6 days of culture of naive blood B cells after stimulation with CpG‐ODN or R848 in combination with T‐cell‐independent B cell class switch inducing factors. Data shown as mean +SEM, *n* = 3–5 donors per group, combined graph of two separate experiments, Mann–Whitney test *p<0.05, **p<0.01, ***p<0.001.

Next, we studied which factors promoted expression of CD38; an activation marker and early marker for plasma cells. Although BAFF in combination with CpG‐ODN or R848 is able to enhance the viability of the cells, it did not enhance CD38 expression compared to medium control (Fig. 2). Retinoic acid strongly induced CD38^+^ cells in total peripheral B cells, as has been reported previously for tonsillar derived total B cells 24. Interestingly, RA also increased CD38^+^ after stimulation of naive B cells for 6 days. This RA‐mediated increase of the percentage of CD38^+^ on total cultured B cells was further enhanced upon addition of CpG‐ODN or R848, while it was only enhanced upon addition of CpG‐ODN in the naive cultured B cells. Interestingly, addition of other cells (B cell‐depleted PBMCs) from the start of the culture induced a dose‐dependent increase of CD38^+^ percentage in cells exposed to medium, APRIL, BAFF and IL‐10, but not to RA (Supporting Information Fig. 3). Addition of APRIL or IL‐10 alone or in combination with CpG‐ODN or R848 to total peripheral blood B cells did not enhance the percentage of CD38^+^ B cells after 6 days (Fig. 2B).

Next, we investigated whether the increased percentage of CD38^+^ B cells in naive cells stimulated with RA for 6 days was also accompanied by CD138 expression (marking short‐ and long‐lived plasmablasts). Although RA alone or in combination with CpG‐ODN or R848 increased the percentage of CD38^+^ B cells in naive B cells cultured for 6 days we did not observe co‐expression with CD138. Overall the percentages of CD38^+^CD138^+^ were very low (<1.5%) in all groups (data not shown).

### Homing marker expression on stimulated total and naive peripheral blood B cells

To allow maximal efficiency, targeting of the antigen‐stimulated cells to the right tissue is important. At the time of activation, factors present in the surrounding tissue can induce expression of homing markers that enable cells to bind to selectins or respond to chemokines that are present at their target tissue. Here we investigated whether APRIL, BAFF, IL‐10 or RA in combination with CpG‐ODN or R848 changed integrin β7 (gut homing; α4β7) or CCR10 (skin, airway and gut homing) expression on B cells after 6 days of culture (Supporting Information Fig. 4 for gating strategy).

**Figure 3:**
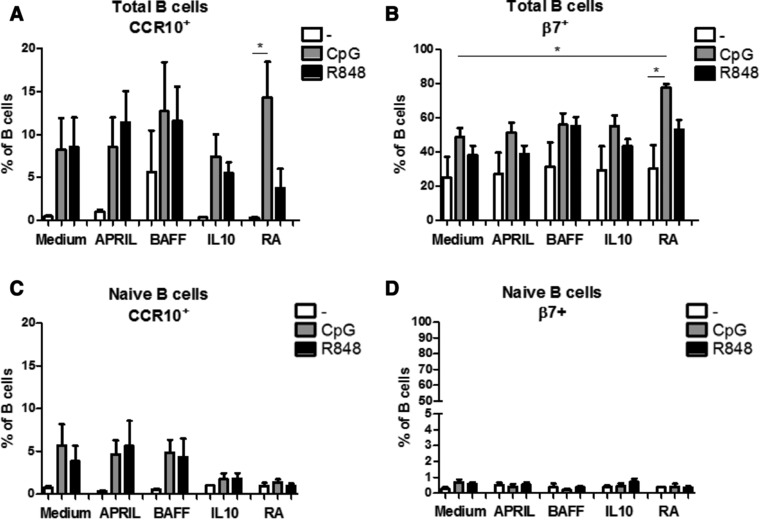
Retinoic acid affects homing marker expression on mature CpG‐ODN stimulated B cells, Percentages of homing molecules CCR10^+^ (A,C) and β7^+^ (B,D) on total peripheral blood (A,B) and naïve (C,D) B cells after 6 days culture with T cell independent B cell class switch inducing factors in combination with CpG‐ODN or R848 as measured by flow cytometry. Data shown as mean + SEM, A and B: *n* = 5 donors per group, combined graph of three separate experiments, Repeated measures ANOVA, Tukey post‐hoc test, *p<0.05, **p<0.01, ***p<0.001, C and D: 3–5 donors per group, combined graph of two separate experiments, *p<0.05, **p<0.01, ***p<0.001 Mann–Whitney test.

The percentage of CCR10^+^ B cells was very variable irrespective of the factor added for both total and naive B cells. Only RA combined with CpG‐ODN increased the percentage of CCR10^+^ B cells in the total B cell experiment compared to adding RA alone (Fig. 3A). After culturing of naïve B cells for 6 days the percentages of CCR10^+^ B cells were lower (compared to total B cells), and no differences were observed between the different factors and stimulants added (Fig. 3C).

Stimulation of the total pool of peripheral blood B cells with CpG‐ODN significantly increased the percentage of β7^+^ B cells in the RA exposed cells. Furthermore, RA augmented the effect of CpG‐ODN significantly compared to CpG‐ODN alone (Fig. 3B). In contrast to the total B cell pool, stimulation of naïve B cells with the different factors in the absence or presence of CpG‐ODN or R848 did not induce higher percentages of β7^+^ B cells (Fig. 3D).

### R848 and CpG‐ODN stimulated total peripheral B cells produce high levels of IgA1 and IgA2

**Figure 4:**
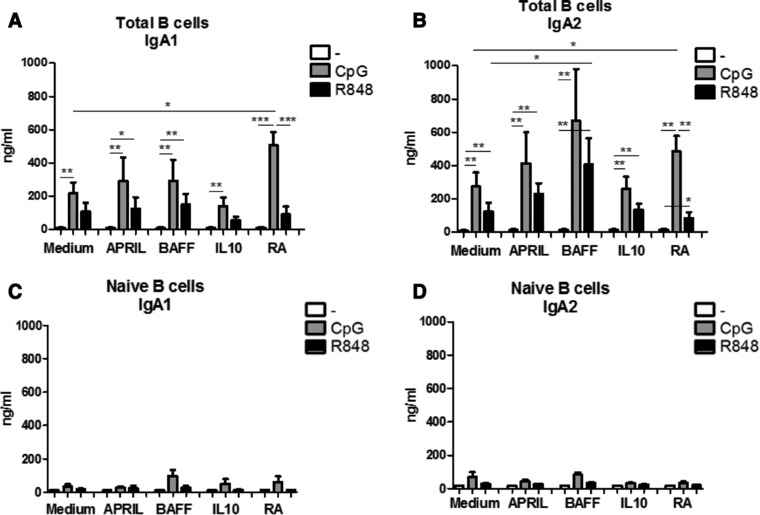
BAFF and Retinoic acid differentially affect IgA1 and IgA2 production in mature stimulated B cells. IgA1 (A,C) and IgA2 (B,D) production by total peripheral blood (A,B) or naïve (C,D) B cells left unstimulated or stimulated for 6 days by CpG‐ODN or R848 in combination with T cell independent B cell class switch inducing factors as measured by IgA1 and IgA2 specific ELISA. Data shown as mean + SEM, A and B: *n* = 8–14 donors per group, combined graph of five separate experiments *p<0.05, **p<0.01, ***p<0.001 Mann–Whitney test, C and D: 3–5 donors per group, combined graph of two separate experiments *p<0.05, **p<0.01, ***p<0.001 Mann–Whitney test.

It was previously reported that T cell‐independent B cell class switch inducing factors such as APRIL and retinoic acid are potent inducers of IgA when co‐stimulated with IL‐10 4, 25. Highly purified unstimulated total peripheral blood B cells (>98%, T cells <1%) did not produce IgA1 or IgA2 after exposure to APRIL, BAFF, IL‐10 or RA (∼10 ± 1.5 ng/ml IgA1, 10 ± 2.1 ng/ml IgA2) (Fig. 4A and B). Similarly, the combination of BAFF +IL‐10 or APRIL + IL‐10 did not induce IgA1 or IgA2 production (data not shown). Stimulation with either CpG‐ODN or R848 induced IgA1 and IgA2 production compared to unstimulated cells (Fig. 4A and B). Retinoic acid enhanced production of IgA1 in CpG‐ODN but not in R848 stimulated B cells (Fig. 4A). The production of IgA1 in R848 stimulated B cells was not influenced by the factors analysed. In contrast, RA augmented IgA2 production in CpG‐ODN stimulated cells while BAFF enhanced IgA2 production in R848 stimulated cells (Fig. 4B).

Stimulation of naïve peripheral blood B cells for 6 days showed that next to lower total levels of IgA1 and IgA2, the combination of BAFF with CpG‐ODN resulted in higher levels of IgA1 compared to CpG‐ODN alone, however this was not significant (Fig. 4C and D). Here we did not observe the higher production of IgA2 in naïve cells stimulated with BAFF and R848 or the effect of RA and CpG‐ODN (Fig. 4D).

Addition of other cells (B cell depleted PMBCs) at the start of the culture dose‐dependently increases IgA1 but not IgA2 production, with the exception of cells exposed to retinoic acid (Supporting Information Fig. 5A). Correlation analysis between the percentage of other cells and IgA1 or IgA2 production clearly showed that the production of IgA1 was correlated to the presence of other cells than B cells at the start of the culture (Supporting Information Fig. 5B). In contrast, for IgA2 this correlation does not exist. Analysis of other immunoglobulin isotypes revealed that BAFF did not augment IgG or IgM production in CpG‐ODN or R848 stimulated total B cells (Supporting Information Fig. 6A and B), while it did augment IgA2 production in R848‐stimulated cells significantly. When naïve B cells were cultured for 6 days increased IgM levels were observed after CpG‐ODN compared to unstimulated cells, and not so much after R848 stimulation for 6 days irrespective of the factor added. For IgG, we observe very low levels (<50 ng/mL) (Supporting Information Fig. 6C and D).

### BAFF augments IL‐10 production by R848 stimulated B cells

**Figure 5:**
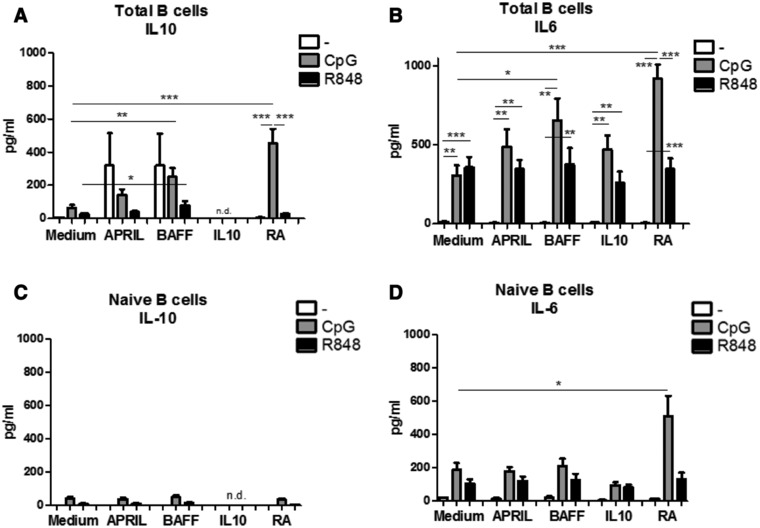
BAFF and Retinoic acid differentially affect IL‐10 and IL‐6 production. IL‐10 (A, C) and IL‐6 (B,D) production by total peripheral blood (A,B) or naïve (C,D) B cells left unstimulated or stimulated for 6 days by CpG‐ODN or R848 in combination with T‐cell independent B‐cell class switch inducing factors as measured by Cytometric Bead Array on a flow cytometer in the supernatant of 6‐day‐old cultures. Data shown as mean + SEM, A and B: *n* = 8–14 donors per group, combined graph of five separate experiments, C and D: 3–5 donors per group, combined graph of two separate experiments *p<0.05, **p<0.01, ***p<0.001 Mann–Whitney test.

Besides their important role as antibody producing cells, B cells have also been shown to secrete cytokines. For example, IL‐6 can be secreted by effector B cells, while IL‐10 can be the hallmark of regulatory B cells 26. In line with a previous study by Yehudai and colleagues 27, we show that BAFF and RA significantly increased IL‐10 and IL‐6 production in CpG‐ODN stimulated B cells compared to CpG‐ODN alone (Fig. 5).

BAFF or APRIL increased IL‐10 production in unstimulated total B cells although this is not significantly different from the medium control (BAFF: *p* = 0.09; APRIL: *p* = 0.33, Fig. 5A). BAFF exposure combined with R848 stimulation induced a small but significant increase in IL‐10 production (from 24 ± 15 to 64 ± 48 pg/mL, Fig. 5A), while BAFF combined with CpG‐ODN enhanced IL‐10 production from 54 ± 15 pg/mL in CPG‐ODN alone to 253 ± 68 pg/mL in BAFF + CpG‐ODN. To investigate whether this IL‐10 production resulted from newly activated naïve B cells, we isolated naïve B cells and exposed them to APRIL, BAFF, or RA in absence or presence of CpG‐ODN or R848. Exposure of naive B cells to BAFF combined with R848 or CpG‐ODN did not result in increased IL‐10 production (Fig. 5C), indicating that mature B cells were responsible for the secretion of IL‐10 seen when stimulating total peripheral blood B cells.

TLR7/8 stimulation by R848 increased IL‐6 production independent of exposure to T cell‐independent B cell class switch factor (Fig. 5B). Likewise, CpG‐ODN alone induced IL‐6 production significantly, and BAFF and RA augmented this production even further (from ∼400 pg/mL to ∼650 or 800 pg/mL, respectively) (Fig. 5B). The significantly higher production of IL‐6 by total B cells stimulated with BAFF and CpG‐ODN was not observed in naïve B cells stimulated with BAFF and CpG‐ODN (Fig. 5D). However, stimulation of both total peripheral blood B cells and naïve B cells with CpG‐ODN in the presence of RA resulted in significantly increased IL‐6 production (Fig. 5D), indicating that the combination of BAFF and CpG‐ODN only increased IL‐6 production by mature B cells, while RA targets both naïve and mature B cells.

## Discussion

In this study we investigated whether different T cell independent B cell conditioning factors are able to boost IgA2 antibody and cytokine production by TLR9 (bacterial) and TLR7/8 (virally) stimulated total and naive peripheral blood B cells. We showed that the TNF‐α family member ‘B cell activating factor’ (BAFF) significantly increased IgA2 and IL‐10 production but not IL‐6 production by TLR7/8 (R848) stimulated highly pure (<98 %) total B cells. Additionally, in CpG‐ODN stimulated total peripheral blood B cells, BAFF significantly increased IL‐10 but also IL‐6 production, indicating a more general activation of those cells. These effects were not observed when naive B cells were cultured in the presence of BAFF and R848 or CpG‐ODN. Our results show that naïve B cells isolated from peripheral blood may respond differently to TI class switch factors than experienced cells isolated from peripheral blood.

It has been known for several years that B cell receptor expression is not the only factor important for B cell survival 28. BAFF and BAFF signalling are important for B cell maturation and can replace the role of CD40‐CD40L interaction in T‐cell independent stimulation 29. In our experiments we did not observe effects of BAFF on viability in unstimulated B cells. However, addition of other cells than B cells (B cell depleted PBMCs) greatly increases viability. We have observed that even a contamination of 2% of T cells present at the start of our cultures significantly increased cell viability and IgA1 production by B cells, so investigating B cells in the T‐independent context requires high levels of purity (<1% T cell contamination) of the starting cell preparation. In addition, cell culture conditions may need further optimization for highly purified B cell assays.

Bacterial and viral ligands are abundantly present on mucosal surfaces that are characterized by the production of high levels of IgA. Currently, data showing how these factors influence the production of IgA subclasses by human B cells are limited. In this study, both R848 and CpG‐ODN stimulated B cells produced high levels of IgA1 and IgA2 compared to unstimulated cells, however these cells are derived from peripheral blood and not mucosal tissue. It is known that CpG‐ODN is able to induce antibody production and especially can drive human transitional and memory B cells to terminal differentiation and stimulate natural antibody (IgM) and matured antibody secretion 30, 31. Here, we observe that the combination of BAFF with R848 is specifically inducing IgA2 production in total peripheral blood, but not naive, B cells. IgM and IgG levels in total peripheral blood B cells do not significantly differ from medium controls after 6 days of culture in the presence of BAFF and R848. When naive B cells were isolated we did not observe this increase in IgA2 production by BAFF in combination with R848, suggesting that BAFF acts on already differentiated (class switched) B cells. Consequently, we suggest that the conditions tested here may enhance antibody production by experienced cells but not induce class switch recombination and antibody production by naïve B cells.

Retinoic acid enhanced both IgA1 and IgA2 production in CpG‐ODN stimulated B cells, which can be directly correlated to the massive induction of the percentage of CD38^+^ B cells, also reported previously 24. BAFF, however, while it did not increase the percentage of CD38^+^ B cells, increased IgA2 production in R848 stimulated total B cells. This may be caused by enhanced viability of B cells or a higher production per individual cell in the presence of BAFF.

Depending on the location of the B cell and the differentiation stage, different receptors for BAFF and APRIL are expressed on B cells, of which some bind to both APRIL and BAFF (TACI, BCMA), while others bind with higher affinity to APRIL (BCMA) or BAFF (BAFF‐R) 32, 33. While we do see high BAFF‐R expression on both naive and memory cells we do not observe high percentages of BCMA^+^ cells. We hypothesize that our results are mainly dependent on BAFF‐R stimulation by BAFF, also since we observed a clear downregulation after 6 days of culture. However, future receptor blocking experiments are warranted to proof this. Previous reports have shown that naïve (IgD^+^) B cells isolated from mucosal sites cultured in the presence of APRIL resulted production of IgA2 4, 31. In this study we did not observe significant induction of IgA2 in naive B cells cultured for 6 days in the presence of APRIL (or BAFF, RA or IL‐10). Assuming similar purity of the B cell preparations, these data suggests that mucosal and blood derived B cells used, while both naïve, are different. Therefore, naïve cells isolated from blood may not accurately represent the response of mucosal B cells even when additional mucosal niche factors are provided. The differences in expression of receptors for BAFF and APRIL under the influence of the local environment could be responsible for such differences.

For the induced plasma cell secretion of IgA2 to be effective at the sites where the antigens are encountered, B cells need to express so‐called homing molecules to be able to migrate to the correct tissues. Retinoic acid has been reported to induce small intestinal homing (CCR9^+^ Integrin‐β7^+^) on a number of different immune cells such as dendritic cells, T and B cells 34, 35, 36, 37, 38. Not much is known about the induction of CCR10^+^ by retinoic acid and more in depth flow cytometric and *ex vivo* analysis is needed to confirm the link between combinations of homing markers and subsequent homing of stimulated B cells to different tissues 39. Our data confirm that retinoic acid is an important factor in regulating the expression of homing markers on mature B cells in the context of TLR stimulation.

Apart from their very important function of antibody secretion, B cells can also modify the immune response by secreting cytokines. Here, we observed that BAFF and retinoic acid significantly enhance IL‐10 and IL‐6 production by CpG‐ODN stimulated B cells, suggesting a general activation response of the B cells. Interestingly, in R848 stimulated B cells, BAFF but not RA showed a small but significantly increase in IL‐10 production, while it did not change IL‐6 production. We did not observe this effect of BAFF and R848 in naïve B cells, indicating that the IL‐10 is mainly derived from mature B cells, which may be confirmed by intracellular flow cytometric analysis in future studies. The small increase of IL‐10, without induction of IL‐6 combined with the induction of IgA2 suggests that in the conditions used in this study stimulation of TLR7/8 in the presence of BAFF results in a more tolerogenic environment which might counteract overt inflammatory responses. Interestingly, BAFF is also found in breast milk during the 6‐month post‐partum period in healthy women and in amniotic fluid 40. Arguing against a protective role for BAFF in (peripheral blood or mucosal sites) is the fact that BAFF has been implicated in auto‐immune disease 41 and increased levels of BAFF in airway epithelial cells have been observed in asthmatic patients. These studies indicate that BAFF may have various functions in different contexts 42. Next to this, viruses also produce IL‐10 like molecules to dampen the immune response and facilitate infection, so the fact that IL‐10 is induced might not be beneficial to the host when an actual virus infection is ongoing 43.

In conclusion, we show that addition of B cell activating factor (BAFF) to TLR7/8 (R848) stimulated B cells augments both IgA2 and IL‐10 production in mature B cells isolated from blood. These data, together with the fact that BAFF is present in amniotic fluid, breastmilk and increased in autoimmune disease and asthma, warrants further investigation of its role in immune regulation both in the periphery and mucosal tissues in early life or during disease.

## Materials and methods

### PBMC and B cell isolation

Peripheral blood mononuclear cells (PBMCs) were obtained from buffy coats of healthy blood donors that provided informed consent (Sanquin Blood Bank, Nijmegen, The Netherlands). Blood was diluted 1:1 in IMDM (12440053, Gibco‐BRL) and isolated by gradient centrifugation on Ficoll‐Paque PLUS (GE Health, Sigma‐Aldrich, GE17‐1440‐02) for 5 min at 200 × *g* and subsequently for 15 min at 500 × *g* (without brake, 20°C). The PBMCs were harvested from the Ficoll layer, resuspended and washed two times in IMDM.

The isolation of the B‐cells was performed with the EasySep™ Human B Cell Enrichment Kit (#19054, Stem Cell Technologies) or the EasySep™ Naïve Human B Cell Enrichment Kit (#19254, Stem Cell Technologies) by negative cell selection at room temperature according to the manufacturer's protocol. To obtain maximal purity the total negative fraction was incubated on the magnet for an additional 5 min to allow removal of magnetic‐bead‐bound‐cells from the negative fraction. Purity of the negative fraction was determined by staining the cells with CD3‐FITC (BW264/56, #130‐080‐401, Miltenyi Biotec) and CD19‐APC (HIB19, #302212, BioLegend) followed by flow cytometric analysis (FACS Canto II BD Biosciences). After this selection a 98–99% pure B cell fraction was obtained. We only considered samples that had a T cell contamination of <1% to be T cell independent samples. In the experiments were we assessed T cell dependent and/or CD40L‐dependent effects on B cells we added the B cell depleted PBMC fraction (200 000 or 500 000 cells per well).

### B cell cultures

B cells were cultured at a concentration of 0.5 million cells/mL in RPMI supplemented with 10% FBS and 1% pen/strep. Stimuli were added and incubated with the cells for 6 days at 37°C in a humidified environment and 5% CO_2_. Stimuli were used as follows (end concentrations): All‐trans RA 10^−5^M (#R2625, Sigma Aldrich), 20 ng/mL IL‐10 (200‐10, Peprotech), 125 ng/mL APRIL (310‐10C, Peprotech) and BAFF (310‐13, Peprotech). CpG‐ODN (ODN‐2006,1.0 μg/mL, Hycult Biotech, #HC4039) or R848 (1.0 μg/mL, Invivogen, #tlrl‐r848) were added to the culture from the start. Cell culture supernatants were collected and stored at ‐20°C until use. Cells were harvested, centrifuged and stained for flow cytometric analysis. Prior to these experiments optimal dose of CpG‐ODN, R848, APRIL, BAFF and RA were assessed. The use of 500 ng/mL APRIL and BAFF 4 did not show different effects compared to the 125 ng/mL used in this study. For CpG and R848 we performed pilot experiments (CpG 1.5, 1, 0.5 and 0.1 ug/mL; R848 3, 1, 0.5 and 0.1 ug/mL. At 1 ug/mL the effect was not different from the higher dose and gave a B cell response on CD38, IL6 and IL‐10 production. For RA we used 10^−8^ to 10^−3^ M, and on the basis of viability of the cells (10^−3^ reduced viability greatly) we choose 10^−5^ M as our dose, since here we observed maximal effects on CD38. Furthermore, IL‐10 concentration of 20 ng/mL was based on a study by Hummelshoj 44 where 10 and 100 ng/mL was used on isolated human B cells and showed the same effect on B cell Ig‐production.

### IgA1 and IgA2 ELISA

ELISA plates (# 650‐061, Greiner Bio One) were coated overnight with 1.0 μg/mL Polyclonal goat anti human IgA (Southern Biotech, 2050‐01). The next day, plates were washed (0.05% tween in PBS) once and blocked with 5% non‐fat dry milk powder (Friesche Vlag). After blocking, plates were washed three times and the 50 μL undiluted cell culture supernatants were added to 50 μL blocking buffer (non‐fat dry milk) per well. The IgA1 and IgA2 recombinant proteins (16‐16‐090701‐1M and 16‐16‐090701‐2M, Athens Research) were serially diluted in blocking buffer to obtain a standard curve. Plates were incubated for 90 min at 37°C. Plates were washed and incubated with 1:5000 biotinylated anti‐IgA1 (#9130‐08, Southern Biotech) or 1:2000 biotinylated anti‐IgA2 (#9140‐08, Southern Biotech) for 1 hour at room temperature while shaking (180 rpm). Plates were washed 6 times and 1:10 000 streptavidin poly HRP (#21140, Thermo Fisher Scientific) was added and incubated for 1 h at room temperature while shaking (180 rpm). 100 μL TMB (#37574 Thermo Fisher Scientific) was added. Plates were developed, subsequently stopped with 2% HCL and measured at 450 nm with 620 nm as reference.

### Flow cytometric analysis

The cells were stained with: CD3‐FITC (BW264/56, #130‐080‐401, Miltenyi Biotec), CD19‐APC (HIB19, #302212, BioLegend), CD38‐PE (IB6, #130‐092‐260, Miltenyi Biotec), CCR10‐APC (REA326, #130‐104‐821, Miltenyi Biotec), β7‐BV421 (FIB504, #564283, BD Bioscience), BCMA‐PE (19F2, #357504, Biolegend), TACI‐APC (1A1, #311912, Biolegend), IL10‐R‐Bv421 (3F9, #742942, BD Biosciences), BAFF‐R‐PE (11C1, #316906, Biolegend), CD138‐APC (44F9, # 130‐098‐746, Miltenyi), and Fixable Viability Dye eFluor 506 (#65‐0866‐14, eBioscience). IL‐6 and IL‐10 levels in supernatants were assessed using the BD Human Flex set (#558276 (IL‐10), #558274 (IL‐6) BD Biosciences) according to the manufacturer's instructions. IgM and IgG in supernatants were assessed using the BD Human Flex set (total IgG #558679 and IgM #558680, BD Biosciences).

### Statistical analysis

Data were visualized and analysed in Prism 5. Flow cytometric data were analysed using FlowJo® v10. Data were tested for normality by Kolmogorov‐Smirnov. Flow cytometry data on total blood B cell cultures were analysed by repeated measures ANOVA after normality testing. Flow cytometry data of naive B cell cultures, antibody and cytokine measurements were tested either non‐parametrically by Mann–Whitney U test or parametrically by Student's *t*‐test. (Non‐ parametric) correlation was analysed by Spearman Rank. Each figure legend indicates the statistics used.

## Conflict of interest

The authors declare no commercial or financial conflict of interest.

**Abbreviations**
APRIL: a proliferation inducing ligand
BAFF: B cell activating factor
BCMA: B‐cell maturation antigen
CSR: class switch recombination
RA: retinoic acid
RSV: respiratory syncytial virus
TACI: Transmembrane activator and CAML interactor
TI: T cell independent

## Supporting information

Peer review correspondence

Supporting Information
