# Supplementary material for: BAFF augments IgA2 and IL‐10 production by TLR7/8 stimulated total peripheral blood B cells
Source: Eur J Immunol. 2017 Oct 11;48(2):283–92. doi: 10.1002/eji.201646861 (PMC5836859; doi:10.1002/eji.201646861)
Supplement: Supplementary file 2 — Supporting Information [file EJI-48-283-s002.pdf]

European Journal of Immunology

Supporting Information

for

DOI 10.1002/eji.201646861

Gerco den Hartog, Thijs L.J. van Osch, Martijn Vos, Ben Meijer, Huub F.J. Savelkoul, R.J. Joost van Neerven and Sylvia Brugman

BAFF augments IgA2 and IL-10 production by TLR7/8 stimulated total peripheral blood B cells

© 2017 The Authors. *European Journal of Immunology* published by WILEY-VCH Verlag GmbH & Co. KGaA, Weinheim.

Supplementary information:

Supplementary figure 1, den Hartog *et al.*

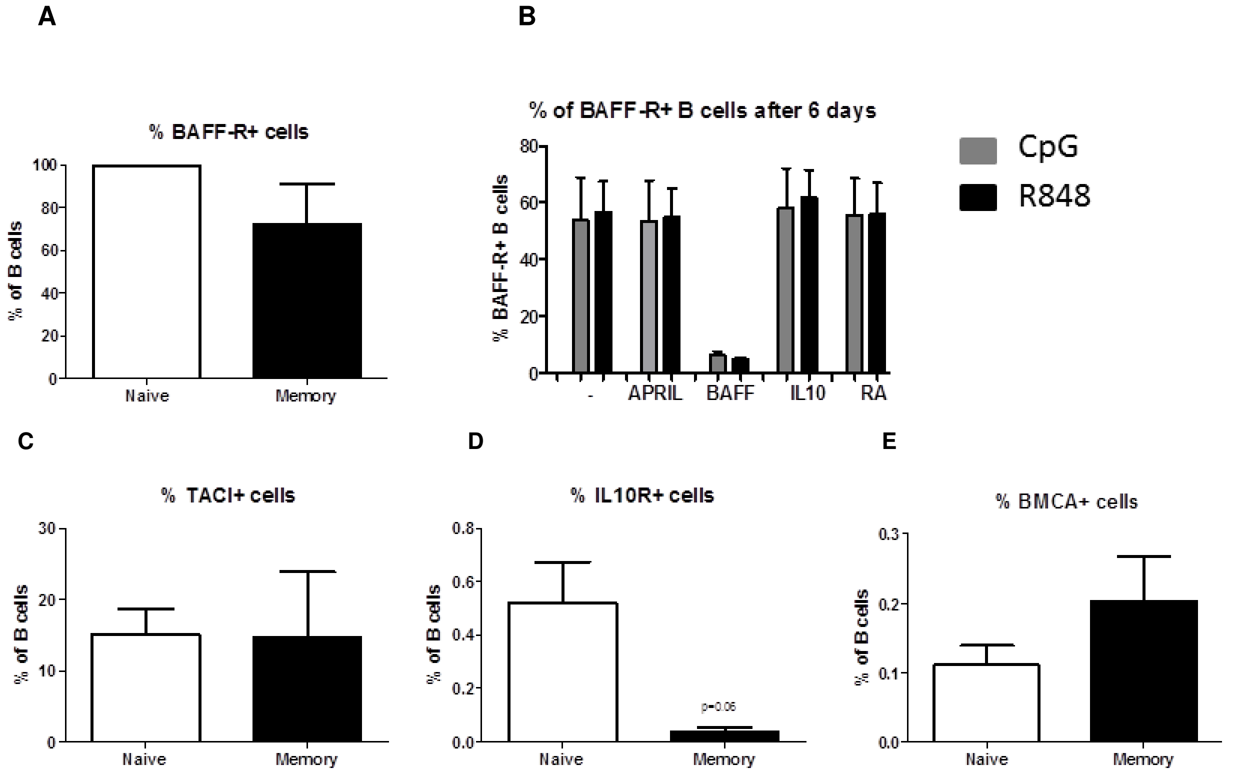

Supplementary figure 1: Receptor expression on naïve versus memory B cells for BAFF (BAFF-R: A, TACI:C, BCMA:E), APRIL (TACI: C, and BCMA:E) and IL-10 (D) at the start of the experiment. The percentages of APRIL, BCMA and IL-10R do not change after 6 days of culture with R848 or CpG-ODN (data not shown), except for BAFF-R for which the percentages go down both in the R848 and CpG-ODN stimulated naïve B cells after 6 days of culture (B). MFI (Geometric mean) shows the same pattern as the percentage graphs for all receptors (data not shown). 2-5 donors per group. The naive B cells have 0.59± 0.09 % CD27 expression. The memory B cells are isolated by first performing total B cell isolation (B cell enrichment kit, Stem cell technologies) followed by CD27-bio labeled antibody combined with streptavidin magnetic beads separation (CD27-bio (130-093-196, Miltenyi), Streptavidin magnetic beads (557812, BD Biosciences).

Supplementary figure 2, den Hartog *et al.*

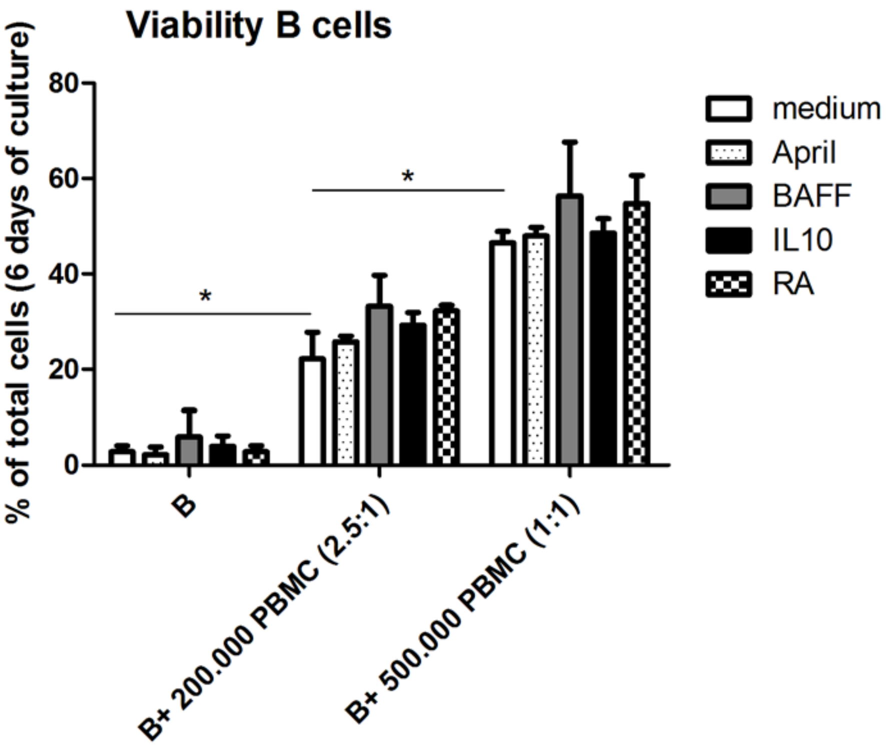

Supplementary figure 2. Percentage of viable B cells after 6 days of culture after stimulation with different mucosal factors in the absence or presence of different amounts of PBMCs devoid of B cells (2-5 donors per group, two separate experiments combined, * $p<0.05$, ** $p<0.01$, *** $p<0.001$ Mann-Whitney test).

Supplemental figure 3, den Hartog *et al.*

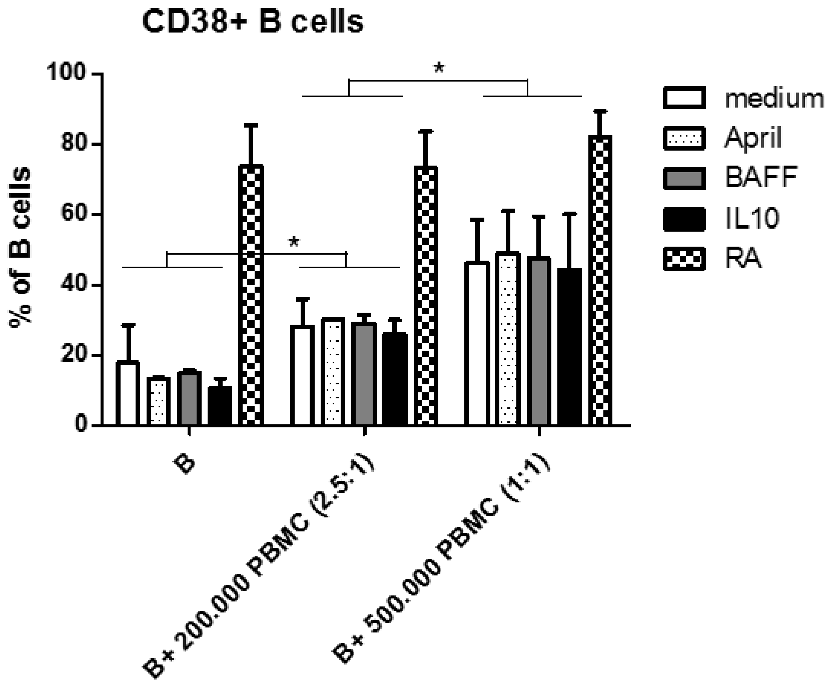

Supplementary figure 3. Percentage of plasma cells after 6 days of culture after stimulation with different mucosal factors in the absence or presence of different amounts of PBMCs devoid of B cells (2-5 donors per group, two separate experiments combined).

Supplementary figure 4, den Hartog *et. al.*

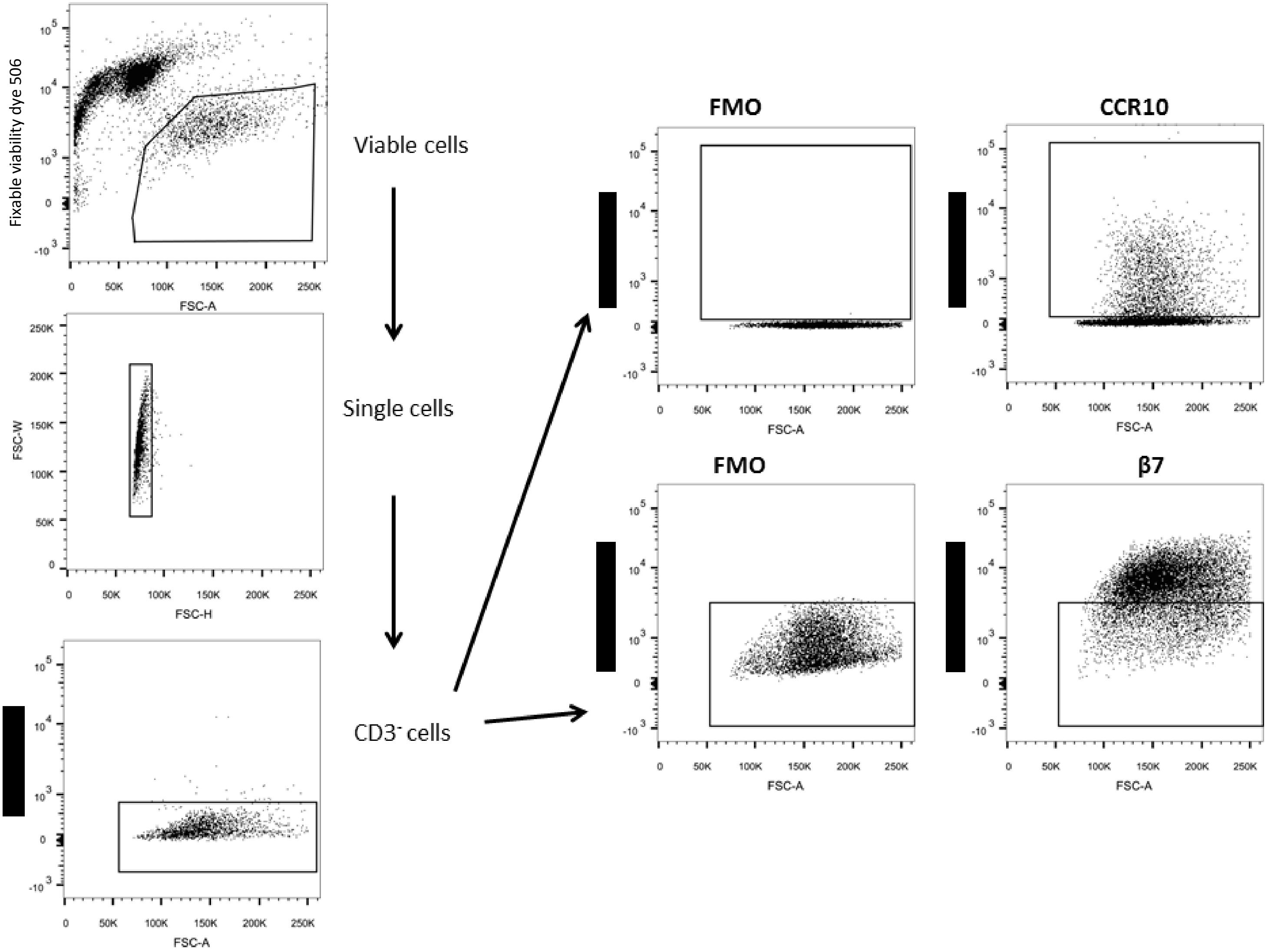

Supplemental figure 4. Gating strategy and example flow cytometry plots to determine the percentages of CCR10+ and Beta7+ B cells.

Supplementary figure 5, den Hartog *et al.*

A

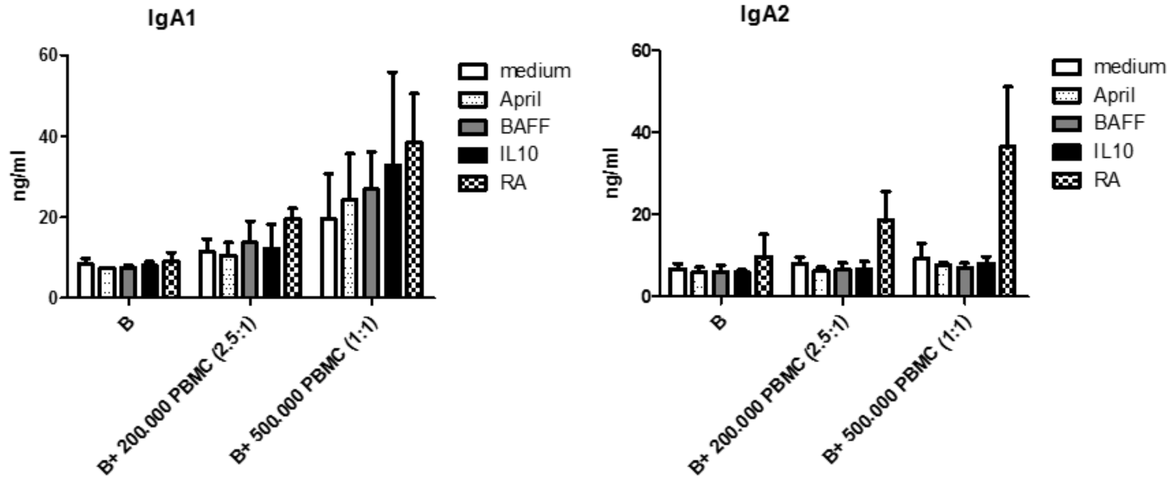

B

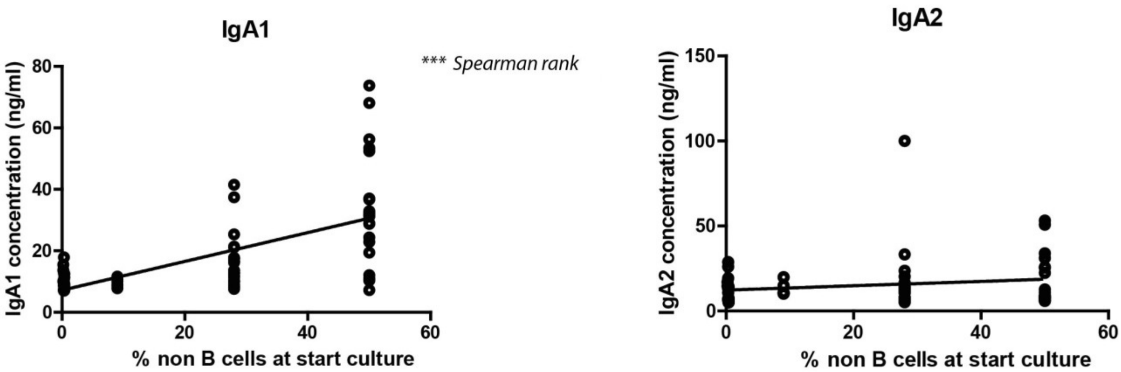

Supplementary figure 5. A:IgA1 and IgA2 production in 6 day B cell cultures after stimulation with different mucosal factors in the absence or presence of different amounts of B cell-depleted PBMCs (2-5 donors per group, two separate experiments combined).B: Correlation between the percentage of non B cells (B cell-depleted PBMCs) present at the start of culture and the production of IgA1 or IgA2 (two experiments combined).

Supplementary figure 6, den Hartog *et al.*

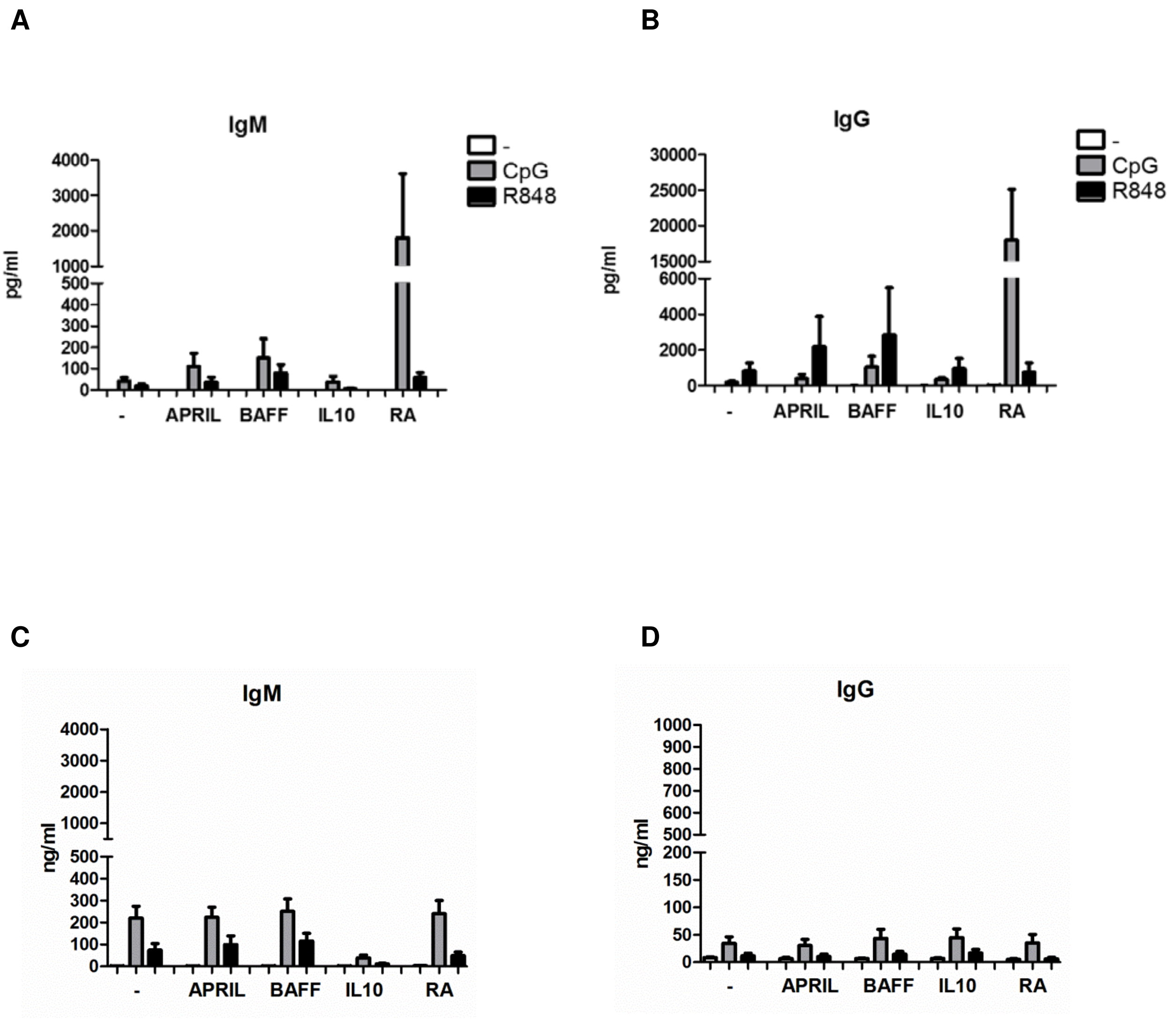

Supplemental figure 6: IgM (A) and IgG (B) production by total B cells after 6 days of culture with medium, APRIL, BAFF, IL-10 or RA in the absence or presence of R848 or CpG-ODN. IgM (C) and IgG (D) production by naive B cells after 6 days of culture with medium, APRIL, BAFF, IL-10 or RA in the absence or presence of R848 or CpG-ODN.
