# Supplementary material for: BAFF augments IgA2 and IL‐10 production by TLR7/8 stimulated total peripheral blood B cells
Source: Eur J Immunol. 2017 Oct 11;48(2):283–92. doi: 10.1002/eji.201646861 (PMC5836859; doi:10.1002/eji.201646861)
Supplement: Supplementary file 1 — Peer review correspondence [file EJI-48-283-s001.pdf]

European Journal of Immunology

BAFF augments IgA2 and IL-10 production by TLR7/8 stimulated total peripheral blood B cells.

Gerco den Hartog, Thijs L.J. van Osch, Martijn Vos, Ben Meijer, Huub F.J. Savelkoul , R.J. Joost van Neerven and Sylvia Brugman

Correspondence: Dr. Sylvia Brugman, Cell Biology and Immunology group, Animal Sciences, Wageningen University, De Elst 1, Room Ee1261, 6708 WD, the Netherlands

---

Review Timeline:	Submission date:	07-Dec-2016
	First Editorial decision:	16-Jan-2017
	First revision received:	31-May-2017;
	Second Editorial decision:	29-Jun-2017
	Second revision received:	31-Aug-2017
	Accepted:	12-Sept-2017

Handling Executive Committee member: Prof Andreas Radbruch

Please note that the correspondence below does not include the standard editorial instructions regarding preparation and submission of revised manuscripts, only the scientific revisions requested and addressed.

First Editorial Decision

29-Jun-2017

Dear Dr. Brugman,

Manuscript ID eji.201646861 entitled "B cell activating factor (BAFF) augments IgA2 and IL-10 production by human B cells upon TLR7/8 stimulation." which you submitted to the European Journal of Immunology has been reviewed. The comments of the referees are included at the bottom of this letter.

A revised version of your manuscript that takes into account the comments of the referees will be reconsidered for publication. Should you disagree with any of the referees' concerns, you should address this in your point-by-point response and provide solid scientific reasons for why you will not make the requested changes.

You should also pay close attention to the editorial comments included below. **In particular, please edit your figure legends to follow Journal standards as outlined in the editorial comments. Failure to do this will result in delays in the re-review process.**

Please note that submitting a revision of your manuscript does not guarantee eventual acceptance, and that your revision will be re-reviewed by the referees before a decision is rendered.

If the revision of the paper is expected to take more than three months, please inform the editorial office. Revisions taking longer than six months may be assessed by new referee(s) to ensure the relevance and timeliness of the data.

Once again, thank you for submitting your manuscript to European Journal of Immunology and we look forward to receiving your revision.

Yours sincerely,
Laura Soto Vazquez

On behalf of Prof. Andreas Radbruch

Editorial Office
European Journal of Immunology
e-mail: ejied@wiley.com
www.eji-journal.eu

****

Reviewer: 1

European Journal of Immunology

Comments to the Author

The manuscript reports BAFF-induced increase in IgA2 secretion and survival of human B cells activated with CpG or R848. The authors propose that BAFF can be used to improve humoral immune responses in breast-fed babies.
Unfortunately, the manuscript is rather incomplete as it lacks essential controls needed for interpreting the data. Moreover, many of the data concerning the function and activity of BAFF, APRIL, RA, CpG and R848 are known. However, the analysis of mucosal B cells from humans has the potential to shed more light on the activity and role of these factors. Therefore, it would be interesting if the authors would concentrate on analyzing these cells.

General comments:
1) The expression "mucosal factors" for BAFF, APRIL, IL-10 and retinoic acid is simply wrong and makes no sense at all because all four factors are also found outside of the mucosa. Moreover, only BAFF and APRIL are related factors, IL-10 and RA are completely unrelated to BAFF/APRIL and to each other. Mucins, lactoferrin and β-defensins are typically "mucosal factors" but not BAFF, APRIL, IL-10 or RA.

2) The authors used circulating B cells from blood throughout their analysis. These cells include transitional (T1/2), follicular (FO), marginal zone-like (MZ), switched memory (mem) B cells and plasma cells (PC). Because of the differential expression patterns of the respective receptors for BAFF, APRIL, IL-10 and RA, these subsets are expected to respond differently to BAFF, APRIL, IL-10 and vitamin A/RA. The authors should therefore repeat their study using B cells that were separated into naive and memory B cells. They also should analyze the expression of BAFFR, TACI, IL-10R and RARα, RARβ, AND RARγ in these different B cells subsets. Without this information, the interpretation of the data is difficult as it remains unclear, which of the subsets actually responds to BAFF, CpG, RA, and R848. In addition, the expression of these receptors may change in response to BAFF, APRIL, CpG and RA.

3) Circulating B cells are not a representative for mucosal B cells. As many mucosal B cells are tissue resident cells, authors should compare their data to tonsillar B cells or B cells from appendices as these sources are more representative for mucosal B cells.

European Journal of Immunology

4) The figures should be labeled appropriately. The lettering copied from the FACS plots cannot be deciphered at all. The figure legends are extemely cryptic and should therefore be completed by providing the concentrations of added cytokines and TLR ligands.

5) The authors add B cell-depleted PBMC ("other cells") to support B cell survival and IgA secretion. This fraction represents a mixture of many different cell types. To mimic the support by T-helper cells, the authors should add purified memory T cells (CD4 RO+ CD4+). If they want to address the role of other cell types they should add either FACS or MACS purified cells.

Specific comments:

Figure 1B shows that the percentage of viable cells even decreases without adding CpG or R848 in the presence of BAFF (about 6% of viable cells) compared to control cells (10% viable cells). This needs to be clarified as BAFF is known to support and not to prevent the survival of B cells. The addition of B cell depleted cells seems to increase the percentage of viable cells (Suppl. Figure 1). It is not clear from this figure to which extent B cells were depleted. A small contamination of B cells could be the cause for the observed increase in viable cells. The authors should exclude this possibility by providing CD19 staining of the B cell-depleted PBMC.

Figure 2 and results: the authors use CD38 as a marker for "plasma cells". Short-and long-lived plasma cells are Blimp-1-positive and express high levels of CD138 and BCMA and low levels or no CD20 and CD19. Although CD38 can be expressed at high levels on plasma cells and plasmablasts as these cells develop from activated resting B cells, increased CD38 expression primarily marks activated B cells. To prove that RA induces development of plasmablasts, the cells should be co-stained for CD19, CD20, CD38, CD138, BCMA and surface IgM, IgG and IgA.

Figure 3. The deviation of CCR10 expression on the surface of BAFF-treated cells is very large – why ? "Bet 7" should be called correctly integrin α4β7.

Figure 4. It is not necessary to label significant differences with "*" where they are obvious, e.g unstimulated cells + BAFF +/- CpG +/- R848. But it would be more interesting to see if cells activated with CpG +/- APRIL BAFF, IL-10 and RA secrete different amounts ot igA1 and IgA2 as it seems that APRIL and RA do not change the secretion of IgA in presence of TLR ligands.

Figure 5. It would be important to know if IL-10 and IL-6 are secreted by naive (IgM+ D+ Cd27-) or by switched memory (IgM- D- CD27+) cells. The authors should repeat the experiment with using B cells fractionated into CD27- and CD27+ cells. If the ELISA is too insensitive they could use intracellular FACS.

Reviewer: 2

Comments to the Author

1. Clarifications

- Either spell out “minutes” or use abbreviation “min” everywhere. Example is in Materials and Methods, page 11, line 297.
- Be consistent in references to companies. Sometime name, city, state and country for the company is indicated and in other cases just the company name and order number. Example: Materials and Methods, page 12, line 324, 325 and 326.
- Write out order number for all substrates/kits used. Especially for BAFF, APRIL and IL-10 in Materials and Methods, page 12, line 325.
- Page 12, line 325. “Both 1 ug/ml CpG-ODN or 1 ug/ml R848 were added to the culture from the start.” I assume that only one of the TLR ligands was added but the word “both” are confusing here.
- The same pattern is used to describe APRIL and RA treated cells in supplemental figures 1, 2 and 4. Change this.

2. FIGURE 1

Figure 1A: You cannot see the label of the Y-axis and only the X-axis very vague.

The text says that stimulation with either CpG-ODN or R848 increases the viability of the cells up to 40% and that it is shown in figure 1 (Results, page 5, line 114-115). However, figure 1B show that the viability of the B cells are around 18-19% with only CpG or R848 compared to 10% in untreated samples. By adding BAFF or RA in combination with CpG the viability increase to 33-35%.

The text says that BAFF significantly increases the viability in combination with either CpG or R848, but Figure 1B only show significance for BAFF + CpG. There is an increase with BAFF and R848 but is it significant? There is no indication in the figure.

There is no mentioning that APRIL or IL-10 alone or in combination with CpG or R848 has no effect on cell viability. I suggest that this result is stated in at least one sentence.

Supplemental figure 1 clearly shows that the presence of other cell types has the most robust effect on viability, suggesting that several factors are necessary for cell survival. Since figure 1B show that the addition of TLR ligands had an effect on the survival of B cells, it would have been interesting if the cells co-cultivated with PBMCs and stimulated with APRIL, BAFF, IL-10 or RA also had been stimulated with CpG or R848. This to as close as possible mimic in vivo.

3. FIGURE 2A: Can't see the Y-axis labeling

4. FIGURE 3: On page 6, line 149-150 it says "In conclusion, stimulation of B cells (by R848 or CpG-ODN) increases the percentage of both CCR10+ B cells and Beta7+ B cells." This sentence is misleading. CpG induces an increase in both CCR10 and Beta7, while R848 only increases the percentage of CCR10+ B cells. This should be clarified.

In addition, page 6, line 151-153 state " Addition of mucosal factors (APRIL, BAFF and IL-10) does not seem to modulate this effect, except for retinoic acid that is able to further enhance the percentage of Beta7+ B cells after CpG-ODN stimulation." This is a repetition, the effect on Beta7 by RA and CpG have already been stated on page 6, line 148-149. However, that RA in combination with CpG increases the expression of CCR10 is not mentioned.

5. FIGURE 4: Page 6, line 162: "This was also not observed for the combination of BAFF or APRIL with IL-10 (data not shown)." What exactly does this mean? That BAFF or APRIL stimulation not induces IL-10 production or that the BAFF or APRIL in combination with IL-10 doesn't induce IgA1 or IgA2 production. It is not clear.
In addition, if the sentence is suppose to say that BAFF + IL-10 or APRIL + IL-10 doesn't induce immunoglobulin production you should say: "similar results were observed when cells were treated with BAFF or APRIL in combination with IL-10 (data not shown).

Figure 4B: The line indicating a significant difference between IgA2 production due to RA+CpG compared

European Journal of Immunology

to CpG alone is not drawn correctly. It indicates a comparison between RA+CpG with R848 alone.

Figure 4B show an increase in IgA2 production due to BAFF and CpG but due to large error bars the difference does not reach significance. However, since the authors later in the discussion state that BAFF increases IgA2 production in combination with CpG or R848 the results should be mentioned here.

6. FIGURE 5: The text describing Figure 5A and 5B are confusing. First the text describes the effect of CpG in combination of mucosal factors on IL-10 and IL-6. Thereafter, the authors' state that APRIL and BAFF increases IL-10 but that the enhancement is not significant. However, only one p-value is shown in Figure 5A. Either show both or none. Then the effect of only R848 on IL-6 is mentioned but that CpG itself has the same effect is left out. Why? Then it goes back to describe the effect of BAFF+R848 on IL-10. I would suggest to state effect of all different stimulation on IL-10 first and thereafter the effect on IL-6.

7. DISCUSSION:

On page 8, line 196 the authors state that "BAFF significantly increased viability of CpG-ODN and R848 stimulated B cells,…." According to the figure, this is not true. Looking at Figure 1B, only the combination of CpG+BAFF significantly increased viability. R848+BAFF also increased viability, but according to the figure the increase is not significant compared to R848 alone.

Page 8, line 202-204: "These experiments show that mucosal factors present at the time of stimulation by TLR ligands can selectively skew the resulting immune response towards IgA." This is not correct. It shows that BAFF in combination with TLR stimulation increase IgA2 production but not IgA1. To conclude that BAFF, or any of the other mucosal factors tested, skew the immune response toward IgA the effect of the factors and TLR stimulation on the production of other antibody isotypes has to be investigated. An IgM and IgG ELISA could be conducted for example.

The discussion lack any mentioning regarding the difference between the effect of APRIL and BAFF. These two factors bind the same receptors and APRIL has been shown before to induce IgA, why not to the same levels as BAFF in this model and under these conditions? Which APRIL molecule was used? In primary B cells APRIL must interact with HSPG to induce signal via TACI for example. There is evidence that TACI expression increases with TLR stimulation (Katsenelson et al 2007), so why are the lower levels APRIL-induced IgA in this study compared to BAFF-induced?

8. Suggestions:

The study focuses on the effect of BAFF, APRIL, IL-10 and RA in combination with TLR ligands on B cell differentiation and IgA production. It would have been interesting to see the effect on the production of other immunoglobulins (IgM for example). In addition, by using flow cytometry or ELISPOT the question if the stimulation increases the production of IgA per B cells or if the stimulation increases the number of cells producing IgA can been answered. RA stimulation increased the percentage of CD38 positive cells, but RA + R848 did not increase IgA production for example. In addition, BAFF had no effect on CD38 expression but BAFF + R848 increased IgA2 production.

Why did not APRIL have the same effect on IgA production as BAFF? Which receptor was responsible for the BAFF mediated IgA production? This can be answered with an experiment using specific receptor inhibitors against BAFFR, TACI and BCMA

Referee #3

This is a study based purely on in vitro observations/approaches, but lacking certain essential details in terms of the dose and time kinetics tested in their experiments which, in my view, are necessary to make valid/meaningful conclusions. In other words, please explain the basis for having chosen the fixed dosages of those various types of cytokines/stimuli used in the cell culture conditions, and also for the timing (Day-6). Did you carry out at least some preliminary experiments to optimize these kinetically? If yes, it needs to be stated or shown as supplementary data.

First revision – authors' response

31-May-2017

First and foremost we would like to thank the reviewers and the editor for their careful review of our manuscript. We appreciate the comments and the suggestions that were instrumental in significantly improving our manuscript. We made substantial changes to the text and performed various additional experiments. Below we respond to each issue raised and indicate what changes were made in the text and what data have been added.

Reviewer: 1

Comments to the Author

The manuscript reports BAFF-induced increase in IgA2 secretion and survival of human B cells activated with CpG or R848. The authors propose that BAFF can be used to improve humoral immune responses in breast-fed babies.
Unfortunately, the manuscript is rather incomplete as it lacks essential controls needed for interpreting the data. Moreover, many of the data concerning the function and activity of BAFF, APRIL, RA, CpG and R848 are known. However, the analysis of mucosal B cells from humans has the potential to shed more light on the activity and role of these factors. Therefore, it would be interesting if the authors would concentrate on analyzing these cells.

General comments:

1) The expression "mucosal factors" for BAFF, APRIL, IL-10 and retinoic acid is simply wrong and makes no sense at all because all four factors are also found outside of the mucosa. Moreover, only BAFF and APRIL are related factors, IL-10 and RA are completely unrelated to BAFF/APRIL and to each other. Mucins, lactoferrin and β-defensins are typically "mucosal factors" but not BAFF, APRIL, IL-10 or RA.

We agree with the reviewer that the term 'mucosal factors' is not completely covering the subject in this context. What we actually meant is 'factors that influence T cell independent B cell responses (resulting in maturation and or Ig production', which are factors such as BAFF, APRIL, IL10 and retinoic acid. These factors are usually present in larger amounts at mucosal sites such as the intestine. We have changed the term 'mucosal factor' in the manuscript to 'T cell independent B cell function promoting factors' to make this more precise.

2) The authors used circulating B cells from blood throughout their analysis. These cells include transitional (T1/2), follicular (FO), marginal zone-like (MZ), switched memory (mem) B cells and plasma cells (PC). Because of the differential expression patterns of the respective receptors for BAFF, APRIL, IL-10 and RA, these subsets are expected to respond differently to BAFF, APRIL, IL-10 and vitamin A/RA. The authors should therefore repeat their study using B cells that were separated into naive and memory B cells. They also should analyze the expression of BAFFR, TACI, IL-10R and RARα, RARβ, AND RARγ in these different B cells subsets. Without this information, the interpretation of the data is difficult as it remains unclear, which of the subsets actually responds to BAFF, CpG, RA, and R848. In addition, the expression of these receptors may change in response to BAFF, APRIL, CpG and RA.
We have used a pool of B cells as to reflect the entire population that could be present in mucosal sites (not only in mucosal lymph nodes, where we could expect more naive cells). We do however agree with the reviewer that more specification of the subsets would give more information into the process of B cell stimulation. Therefore, we have repeated all the experiments with only naive B cells using a negative selection kit, since we feel that this would be more informative on whether naive B cells actually can undergo class-switching in response to the T cell independent factors (APRIL, BAFF,IL-10, or RA) in the presence of CpG or R848 stimulation.

We observed that the viability of the naive cells after 6 days is much lower even after stimulation with CpG or R848 (~5% for the stimulated and around 1% for the unstimulated naive B cells, which is to be expected) (new Figure 1C). For CD38+ expression, although we did observe the same pattern (high % in

RA stimulated cells) as with the total B cell pool, again the percentages of CD38+ B cells were lower when only naive B cells are cultured for 6 days (new figure 2C). As for integrin-β7 expression we clearly see that this marker is hardly present on naive B cells even when they are cultured for 6 days in the presence of different factors and stimulated by CpG or R848 (New figure 3D). The percentages of CCR10+ B cells are lower on the naive B cells after 6 days of culture and here we do not observe an increased percentage in the CpG + RA group which we did observe in the total B cell pool (New figure 3C). As for the IgA1 and IgA2 production; the effect seen on the total B cell pool with BAFF in combination with R848 is not observed when naive B cells are cultured with BAFF and R848. Instead, we observed that BAFF in combination with CpG tended to increase IgA1 production (although not significantly) and IgA2 production did not differ between BAFF and CpG and medium and CpG (New figure 4C and D). When we look into CD138 expression after 6 days of naive B cell culture we observe very low percentages and no significant differences between the different treatments (data not shown). Concerning the IL-10 and IL-6 production: when naive cells were cultured for 6 days the unstimulated cells did not produce any cytokines as expected, however, stimulation of the cells with CpG in the presence of RA increases IL6 production, while no differences are observed for IL-10 in any of the conditions compared to medium control (New figure 5C and D), indicating that the effects of BAFF combined with R848 are due to mature B cells and not newly activated naïve B cells.

Unfortunately, we were not able to investigate RA receptor expression due to the lack of suitable antibodies that could be used on flow cytometry. Although we did consider to investigate expression levels for the different RARs by qPCR, we feel that comparing expression levels would not tell us anything about functionality. Morikawa et al. reported that although resting B cells from tonsils expressed mRNA for all the RA receptors, expression of RARa was the strongest, followed by RXRa, and RARh and RARg were relatively weak (Morikawa, Int. Immunopharmacol. 2005). Furthermore, Seo et al reported that the class-switch induction by RA in human B cells was mediated through RARa (Seo , Human Immunology ,2014). In this study Seo et al used tonsillar B cells (CD20+ B cells). We clearly observe a response to RA in several of our read-outs, future experiments will focus more on the specific pathways of the observed effects.

Furthermore, we have investigated BCMA, BAFFR, TACI and IL10R expression on negatively isolated naive B cells and CD27+ (memory) B cells by flow cytometry. For the naive B cells we also analysed expression of these receptors after 6 days of culture (with CpG and R848) and added this data to the manuscript. Interestingly, a higher percentage of naive B cells was positive for IL10R and BAFF-R compared to the memory B cells. BCMA+ B cells were hardly present at day 0. When looking at the mean fluorescence intensity, there is no difference between naive and memory B cells. When we look into the receptor expression on naive B cells after 6 days of culture (in the presence of CpG and R848) we observe that the percentages (and MFI) of IL10R, BCMA and TACI are comparable between the different groups, however, % of BAFF-R (and MFI) are significantly lower in the BAFF exposed B cells, indicating that either the BAFF-R is internalized upon activation or downregulated as part of a negative feedback loop (New supplementary figure 1).

3) Circulating B cells are not a representative for mucosal B cells. As many mucosal B cells are tissue resident cells, authors should compare their data to tonsillar B cells or B cells from appendices as these sources are more representative for mucosal B cells.

Although we agree with the reviewer that having mucosal B cells to compare with peripheral blood derived B cells would have been great, however, we cannot obtain these samples. In the Netherlands, it is not customary to preventively remove tonsils or appendices anymore (which was done in the past) so it is very rare to get baseline specimens. Therefore, when such mucosal specimens are obtained, this

mainly concerns inflamed mucosal tissue (tonsilitis or appendicitis) and this would greatly interfere with our aim to look at the effect of the factors tested in this study.

4) The figures should be labeled appropriately. The lettering copied from the FACS plots cannot be deciphered at all. The figure legends are extemely cryptic and should therefore be completed by providing the concentrations of added cytokines and TLR ligands.
We apologize for the insufficient quality of our FACS plots and have improved these. We have included the concentrations in the figure legends.

5) The authors add B cell-depleted PBMC ("other cells") to support B cell survival and IgA secretion. This fraction represents a mixture of many different cell types. To mimic the support by T-helper cells, the authors should add purified memory T cells (CD4 RO+ CD4+). If they want to address the role of other cell types they should add either FACS or MACS purified cells.
Our main aim of the study was to investigate whether T cell independent B cell activation and differentiation could be influenced by APRIL, BAFF, IL10 or RA in the context of either bacterial or viral activation. To this end, we chose not to focus on specific T cell-dependent mechanisms. We did however include a graph in the supplementary data showing that if you add other cells in different concentrations IgA1 production is influenced, while IgA2 is not (except for stimulations with RA) . Although we agree that dissecting either the monocyte' or T subset' influence on B cell activation and differentiation would yield interesting results as well this was not our primary research aim.

Specific comments:
Figure 1B shows that the percentage of viable cells even decreases without adding CpG or R848 in the presence of BAFF (about 6% of viable cells) compared to control cells (10% viable cells). This needs to be clarified as BAFF is known to support and not to prevent the survival of B cells.
We have added some text in the results section to specifically address this. In our observations indeed, very pure B cells without any stimulation have a very poor survival even when BAFF is added and although they have BAFF-R. Interestingly, Darce et al (JI 2007) showed that whereas BAFF significantly enhances immunoglobulin secreting cell (ISC) differentiation in response to T cell-dependent activation, BAFF considerably attenuated ISC differentiation of memory B cells in response to CpG stimulation, a form of T cell-independent activation. It might be that this also is reflected in the survival of the cells, but this is pure speculation.

The addition of B cell depleted cells seems to increase the percentage of viable cells (Suppl. Figure 1). It is not clear from this figure to which extent B cells were depleted. A small contamination of B cells could be the cause for the observed increase in viable cells. The authors should exclude this possibility by providing CD19 staining of the B cell-depleted PBMC.
Since we use a negative selection kit to isolate our B cells, staining of the remaining PBMC fraction is not possible, due to the interference of the selection beads. On the basis of the data however using different amounts of PBMCs depleted from B cells we feel that the contribution of contamination of B cells to the viability is unlikely. We use the B cell depleted fraction of PBMCs either 200.000 or 500.000 per well. If we would assume that the PBMC fraction has still 3% of B cells left (which probably is an overestimation, normally B cells account for 1-7% of the PMBCs, and those percentages we see as yield after negative selection), that would yield up to 6.000 B cells extra (for the 200.000) or 15.000 B cells extra (for the 500.000) on top of the 500.000 B cells used. Although we do not have formal proof, these numbers are very unlikely to account for the increase of 20% or 40 % in viability respectively.

Figure 2 and results: the authors use CD38 as a marker for "plasma cells". Short-and long-lived plasma cells are Blimp-1-positive and express high levels of CD138 and BCMA and low levels or no CD20 and CD19. Although CD38 can be expressed at high levels on plasma cells and plasmablasts as these cells develop from activated resting B cells, increased CD38 expression primarily marks activated B cells. To prove that RA induces development of plasmablasts, the cells should be co-stained for CD19, CD20, CD38, CD138, BCMA and surface IgM, IgG and IgA.

Although several other papers use CD38 as a marker for plasma cells, we agree with the reviewer that CD38 is also a marker of recently class-switched B cells (Shapiro-Shelef Nat Rev,Imm 2005). We have changed the text accordingly. To address whether naïve B cells could develop into plasmablasts we have added an additional panel in our new experiments (only naive cells). We have investigated the percentage of CD138+ B cells in the CD38+ fraction after 6 days of culture of naive B cells with different factors and TLR stimulation and did not observe high percentages of CD138+CD38+ (<1,5%).Yet, naïve cells cultured for 6 days do produce antibodies which can only mean that there ISCs is induced. We do not have an explanation for this lack of CD138+ marker. Our naive B cell isolation yielded B cells that had 0.59 ± 0.09 % CD27+ expression. Since we cannot proof that the cells with high CD38+ (in the RA group) are also CD138+ we have changed the text accordingly and speak of activated/recently class-switched and/or plasma cells.

Figure 3. The deviation of CCR10 expression on the surface of BAFF-treated cells is very large – why ? "Bet 7" should be called correctly integrin α4β7.

We cannot answer the question why CCR10 expression on the surface of BAFF-treated cells is very large (not only on BAFF treated cells, but also in the other groups the variability is large). This is usually observed when there are low percentages, as is the case here.
We have changed Beta7 to integrin-β7 or β7+ B cells since we did not use an antibody that co-stains α4.

Figure 4. It is not necessary to label significant differences with "*" where they are obvious, e.g unstimulated cells + BAFF +/- CpG +/- R848. But it would be more interesting to see if cells activated with CpG +/- APRIL BAFF, IL-10 and RA secrete different amounts ot igA1 and IgA2 as it seems that APRIL and RA do not change the secretion of IgA in presence of TLR ligands.

In this graph we have indicated all the significant results. If there is no asterix, it was not significant.

Figure 5. It would be important to know if IL-10 and IL-6 are secreted by naive (IgM+ D+ Cd27-) or by switched memory (IgM- D- CD27+) cells. The authors should repeat the experiment with using B cells fractionated into CD27- and CD27+ cells. If the ELISA is too insensitive they could use intracellular FACS.

We have repeated the entire experiment using naive cells and observed that when naive cells were cultured for 6 days the unstimulated cells did not produce any cytokines (as expected), however, stimulation of the cells with CpG in the presence of RA increases IL-6 production, while no differences are observed for IL-10 in any of the conditions (so also not the BAFF + R848 combination compared to medium control (New figure 5C and D). Therefore, we can conclude that mature B cells are the source of this IL-10 and IL-6 and not so much newly activated naïve B cells.

Reviewer: 2

Comments to the Author

1. Clarifications

- Either spell out “minutes” or use abbreviation “min” everywhere. Example is in Materials and Methods, page 11, line 297.

Changed accordingly.

- Be consistent in references to companies. Sometime name, city, state and country for the company is indicated and in other cases just the company name and order number. Example: Materials and Methods, page 12, line 324, 325 and 326.

Changed accordingly

- Write out order number for all substrates/kits used. Especially for BAFF, APRIL and IL-10 in Materials and Methods, page 12, line 325.

Changed accordingly

- Page 12, line 325. “Both 1 ug/ml CpG-ODN or 1 ug/ml R848 were added to the culture from the start.” I assume that only one of the TLR ligands was added but the word “both” are confusing here.

Indeed, we agree that this is confusing, we have changed the sentence accordingly

- The same pattern is used to describe APRIL and RA treated cells in supplemental figures 1, 2 and 4. Change this.

Changed accordingly

2. FIGURE 1

Figure 1A: You cannot see the label of the Y-axis and only the X-axis very vague.

We agree that the pictures were of insufficient quality for which we apologize, we have changed this accordingly.

The text says that stimulation with either CpG-ODN or R848 increases the viability of the cells up to 40% and that it is shown in figure 1 (Results, page 5, line 114-115). However, figure 1B show that the viability of the B cells are around 18-19% with only CpG or R848 compared to 10% in untreated samples. By adding BAFF or RA in combination with CpG the viability increase to 33-35%.

We agree that this is not an adequate description of the results and have specified this better in the text.

The text says that BAFF significantly increases the viability in combination with either CpG or R848, but Figure 1B only show significance for BAFF + CpG. There is an increase with BAFF and R848 but is it significant? There is no indication in the figure.

Indeed, the viability of BAFF in combination with R848 is also significantly different compared to R848 alone, we have added updated the figure now containing the correct significant comparisons.

There is no mentioning that APRIL or IL-10 alone or in combination with CpG or R848 has no effect on cell viability. I suggest that this result is stated in at least one sentence.

We agree with the reviewer and have added a statement on APRIL and IL-10 in the results section on figure 1.

Supplemental figure 1 clearly shows that the presence of other cell types has the most robust effect on viability, suggesting that several factors are necessary for cell survival. Since figure 1B show that the addition of TLR ligands had an effect on the survival of B cells, it would have been interesting if the cells co-cultivated with PBMCs and stimulated with APRIL, BAFF, IL-10 or RA also had been stimulated with CpG or R848. This to as close as possible mimic in vivo.

We agree with the reviewer that this would have been interesting, however, this was not the focus of our paper. We decided not to expand these experiments, since we were mainly interested in the effect

of the different (T cell independent B cell switch factors/ mucosal) factors on isolated B cells in the absence of other cells.

3. FIGURE 2A: Can't see the Y-axis labeling
We improved the labeling.

4. FIGURE 3: On page 6, line 149-150 it says "In conclusion, stimulation of B cells (by R848 or CpG-ODN) increases the percentage of both CCR10+ B cells and Beta7+ B cells." This sentence is misleading. CpG induces an increase in both CCR10 and Beta7, while R848 only increases the percentage of CCR10+ B cells. This should be clarified.
We have changed this accordingly, indeed this wording does not adequately describe the data.

In addition, page 6, line 151-153 state " Addition of mucosal factors (APRIL, BAFF and IL-10) does not seem to modulate this effect, except for retinoic acid that is able to further enhance the percentage of Beta7+ B cells after CpG-ODN stimulation." This is a repetition, the effect on Beta7 by RA and CpG have already been stated on page 6, line 148-149. However, that RA in combination with CpG increases the expression of CCR10 is not mentioned.
We have changed the text accordingly.

5. FIGURE 4: Page 6, line 162: "This was also not observed for the combination of BAFF or APRIL with IL-10 (data not shown)." What exactly does this mean? That BAFF or APRIL stimulation not induces IL-10 production or that the BAFF or APRIL in combination with IL-10 doesn't induce IgA1 or IgA2 production. It is not clear.
In addition, if the sentence is suppose to say that BAFF + IL-10 or APRIL + IL-10 doesn't induce immunoglobulin production you should say: "similar results were observed when cells were treated with BAFF or APRIL in combination with IL-10 (data not shown).
Indeed, this is the correct interpretation of the sentence and we agree that this was unclear. We have changed the sentence as suggested.

Figure 4B: The line indicating a significant difference between IgA2 production due to RA+CpG compared to CpG alone is not drawn correctly. It indicates a comparison between RA+CpG with R848 alone.
Indeed, it appeared we statistically compared RA+CpG, with R848 alone, which was not correct. We have elongated the line so it correctly indicates the significance between RA+ CpG and CpG alone.

Figure 4B show an increase in IgA2 production due to BAFF and CpG but due to large error bars the difference does not reach significance. However, since the authors later in the discussion state that BAFF increases IgA2 production in combination with CpG or R848 the results should be mentioned here.
We have included additional sentences clarifying this point, and have rewritten the sentence in the discussion so it now indicates that BAFF in combination with R848 increases IgA2 production.

6. FIGURE 5: The text describing Figure 5A and 5B are confusing. First the text describes the effect of CpG in combination of mucosal factors on IL-10 and IL-6. Thereafter, the authors' state that APRIL and BAFF increases IL-10 but that the enhancement is not significant. However, only one p-value is shown in Figure 5A. Either show both or none.
We have removed the p value from the graph

Then the effect of only R848 on IL-6 is mentioned but that CpG itself has the same effect is left out. Why? Then it goes back to describe the effect of BAFF+R848 on IL-10. I would suggest to state effect of all different stimulation on IL-10 first and thereafter the effect on IL-6.

Indeed, we agree that this section is confusing. We have rewritten the text to first describe the effects seen on IL-10 and then continue with the effects on IL-6. We have included a sentence on the effect of CpG on IL-6 production.

7. DISCUSSION:

On page 8, line 196 the authors state that "BAFF significantly increased viability of CpG-ODN and R848 stimulated B cells,…." According to the figure, this is not true. Looking at Figure 1B, only the combination of CpG+BAFF significantly increased viability. R848+BAFF also increased viability, but according to the figure the increase is not significant compared to R848 alone.

We agree with the reviewer and have changed the statement accordingly.

Page 8, line 202-204: "These experiments show that mucosal factors present at the time of stimulation by TLR ligands can selectively skew the resulting immune response towards IgA." This is not correct. It shows that BAFF in combination with TLR stimulation increase IgA2 production but not IgA1. To conclude that BAFF, or any of the other mucosal factors tested, skew the immune response toward IgA the effect of the factors and TLR stimulation on the production of other antibody isotypes has to be investigated. An IgM and IgG ELISA could be conducted for example.

We have now included measurements of IgG and IgM for both total B cells and naive B cells after 6 days of culture. We observed that BAFF and R848 indeed also induced IgG (although not significantly) in the total B cell pool experiments. For the naive B cells we see increased IgM after CpG, and not so much after R848 stimulation for 6 days irrespective of the factor added. For IgG, we observe very low levels (<50 ng/ml)(New Supplementary figure 6). So it does seem that BAFF specifically increases IgA2 and not other isotypes in combination with R848 in mature B cells.

The discussion lack any mentioning regarding the difference between the effect of APRIL and BAFF. These two factors bind the same receptors and APRIL has been shown before to induce IgA, why not to the same levels as BAFF in this model and under these conditions? Which APRIL molecule was used? In primary B cells APRIL must interact with HSPG to induce signal via TACI for example. There is evidence that TACI expression increases with TLR stimulation (Katsenelson et al 2007), so why are the lower levels APRIL-induced IgA in this study compared to BAFF-induced?

Depending on the location of the B cell and the differentiation stage, different receptors for BAFF and APRIL are expressed on B cells, of which some bind to both APRIL and BAFF (TACI), while others bind with higher affinity to APRIL (BCMA) or BAFF (BAFF-R) alone (Bossen, C. Sem in Immunol. 2006 ; Darce, J.R., et al., J. of Immunol. 2007). While we do see high BAFF-R expression both on naive and memory cells we do not observe high percentages of BCMA+ cells (New Supplementary figure 1). We hypothesize that the effects we see are mainly due to BAFF-R stimulation by BAFF (and not APRIL), since we observe a clear downregulation after 6 days of culture. However, we would need additional experiments including receptor blocking assays to really prove this point. This will be subject to our future studies.

8. Suggestions:

The study focuses on the effect of BAFF, APRIL, IL-10 and RA in combination with TLR ligands on B cell differentiation and IgA production. It would have been interesting to see the effect on the production of other immunoglobulins (IgM for example). In addition, by using flow cytometry or ELISPOT the question if the stimulation increases the production of IgA per B cells or if the stimulation increases the number of

cells producing IgA can been answered. RA stimulation increased the percentage of CD38 positive cells, but RA + R848 did not increase IgA production for example. In addition, BAFF had no effect on CD38 expression but BAFF + R848 increased IgA2 production.
We have now included information of other immunoglobulins.

Why did not APRIL have the same effect on IgA production as BAFF? Which receptor was responsible for the BAFF mediated IgA production? This can be answered with an experiment using specific receptor inhibitors against BAFFR, TACI and BCMA
Indeed, we agree with the reviewer, however, we did not choose to perform blocking experiments for this paper but will further dive into the mechanism in our future experiments. We do however observe that BCMA receptor is hardly expressed while BAFF-R is present on both the naïve and memory cells and is downregulated after 6 days of culture in the presence of BAFF.

Referee #3

This is a study based purely on in vitro observations/approaches, but lacking certain essential details in terms of the dose and time kinetics tested in their experiments which, in my view, are necessary to make valid/meaningful conclusions. In other words, please explain the basis for having chosen the fixed dosages of those various types of cytokines/stimuli used in the cell culture conditions, and also for the timing (Day-6). Did you carry out at least some preliminary experiments to optimize these kinetically? If yes, it needs to be stated or shown as supplementary data.
Indeed, we have done several previous experiments in our lab to investigate the different dosages and used the doses of each stimulant accordingly, or used dosages reported in literature to be effective in B cell stimulation experiments. We have included specification of these dosages in the materials and methods including references. In brief, we have tested APRIL and BAFF also in higher concentrations, as reported by He et al. Immunity 2007 (500 ng/ml) and did not observe different effects compared to the 125 ng/ml used in this study. Furthermore, IL-10 concentration of 20 ng/ml was based on a study by Hummelshoj (Scand. J. Immunol. 2006) where 10 and 100 ng/ml was used on isolated human B cells and both concentrations showed the same effect on B cell Ig-production. On the basis of this report, we have chosen 20 ng/ml to perform our experiments. Indeed others have used 50 ng/ml (Xu et al, Nat. Imm. 2007), which might in hindsight could have been used in our experiments, since we did not see much response to 20ng/ml IL-10. For CpG and R848 we performed pilot experiments (CpG 1.5, 1, 0.5 and 0.1 ug/ml; R848 3, 1, 0.5 and 0.1 ug/ml. At 1 ug/ml the effect was not different from the higher dose and gave a B cell response on CD38, IL6 and IL-10 production.
For RA we used 10^{-8} to 10^{-3} M, and on the basis of viability of the cells (10^{-3} is reducing viability) we choose 10^{-5} M as our dose, since here we see maximal effects on CD38.

Second Editorial Decision

29-Jun-2017

Dear Dr. Brugman,

European Journal of Immunology

Thank you for submitting your revised manuscript ID eji.201646861.R1 entitled "BAFF augments IgA2 and IL-10 production by TLR7/8 stimulated total peripheral blood B cells." to the European Journal of Immunology. Your manuscript has been re-reviewed and the comments of the referees are included at the bottom of this letter.

Unfortunately, the referees were not satisfied with the revisions made and further major revision is requested. The journal does not encourage multiple rounds of revision and our Executive Editor encourages you to address the concerns of the referees experimentally in this final round of revision. Should you disagree with any of the referees' concerns, you should address this in your point-by-point response and provide solid scientific reasons for why you will not make the requested changes.

You should also pay close attention to the editorial comments included below. **In particular, please edit your figure legends to follow Journal standards as outlined in the editorial comments. Failure to do this will result in delays in the re-review process.**

Please note that submitting a revision of your manuscript does not guarantee eventual acceptance, and that your revision will be re-reviewed by the referee(s) before a decision is rendered.

If the revision of the paper is expected to take more than three months, please inform the editorial office. Revisions taking longer than six months may be assessed by new referee(s) to ensure the relevance and timeliness of the data.

Once again, thank you for submitting your manuscript to European Journal of Immunology and we look forward to receiving your revision.

Yours sincerely,
Eloho Etemire

On behalf of
Prof. Andreas Radbruch

Dr. Eloho Etemire

Editorial Office

European Journal of Immunology

e-mail: ejied@wiley.com

www.eji-journal.eu

****

Reviewer: 2

Comments to the Author

The study is conducted to address the question if T-cell independent factors BAFF, APRIL, IL-10 and Retinoic acid mediates IgA production in the setting of a viral or bacterial infection. Further, the study specifically investigate immunoglobulin production in naïve and mature B cells to address which subtype that are responsible for the IgA production. Based on the results showing increased IgA2 and IL-10 production by mature B cells due to BAFF + TLR7/8 stimulation and the fact that BAFF is present in breast milk, the authors suggest that BAFF treatment might improve mucosal immunity in infants.

General

The manuscript has improved due to the addition of the new manuscripts and the rewriting. There are still some inconsistency in the language throughout the paper. For example, in several places the word "and" is used where is most likely should say "or". One example is page 14, row 99: "Although BAFF in combination with CpG-ODN and R848…" It should be "…BAFF in combination with CpG-ODN or R848…" since no experiments using CpG-ODN and R848 in combination was conducted. Please go over and change and to or where it is appropriate to reduce confusion.

Labeling of some figures is not enough. For example, the Y-axis in most of the flow cytometry plots are just labeled with the fluorochrome used. It is better and easier to understand the graph if it is labeled with the actually marker measured. In supplemental figure 4 for example, the Y-axis here is indicating CCR10 or beta7 but instead says "APC" or "Pacific Blue" (except one of the graphs which says APC-CCR10).

European Journal of Immunology

Abstract

The wording “viral or bacterial stimulation” should be changed to “viral or bacterial infection.” Same thing with “mimicking viral/bacterial stimulation.” TLR stimulated is correct though.

Results

Figure 1B and 1C: The Y-axis of the two graphs are different even though they show the same thing. In addition, to make it clearer, add “Viability of total B cells” as a header for 1B and “viability of naïve B cells” as header for 1C. This should be done for all the graphs that either look at the total B cells population or the naïve B cells.

Supplemental figure 1. In the text the authors state that they observe IL-10 receptors on both naïve and memory B cells. What is this interpretation based on when according to supplemental figure 1D there are almost no IL-10 receptor positive cells? Especially not in memory B cell population. The percentage of IL-10R in the memory population is as low as the percentage of BMCA expression, which the authors state that they “hardly observe.”

Figure 2A-C. Even though tested, nothing is mentioned regarding the non-existing effect of APRIL or IL-10 on CD38 expression. It should at least be stated in one sentence.

Why was CD138 expression only investigated in the naive B cells population and not among total B cells? Especially when viability, and IgA and cytokine production is the highest.

Page 15, row 116 says “mucosal factor” when in the rest of the manuscript BAFF, APRIL, IL-10 and RA is no longer called mucosal factors.

Figure 5. The test regarding figure 5 is a little confusing. First the authors, correctly, state that BAFF in combination with either R848 or CpG mediate a significant increase in IL-10. Then right after, when looking at naïve B cells, the authors only mention the results for BAFF+R848 stimulation even though BAFF+CpG stimulation support the conclusion they draw (that mature B cells are responsible for the production of IL-10).

Page 16, row 137: “This was also not observed….” This sentence is unclear. If the data (not shown) show that BAFF+IL10 and APRIL+IL-10 does not induce IgA production, it is clearer to write “similar results were observed with…..”

Material and Methods

Page 24, row 280: “ IgM and IgM in supernatant….” Should be “IgM and IgG…”

Reviewer: 1

Comments to the Author
The authors followed most of the suggestions made in the first review. However, some concerns still remain. They should be resolved before publication.

Specific comments
1) In line 55pp of their response the authors state .." Although we agree with the reviewer that having mucosal B cells to compare with peripheral blood erived B cells would have been great, however, we cannot obtain these samples. In the Netherlands, it is not customary to preventively remove tonsils or appendices anymore (which was done in the past) so it is very rare to get baseline specimens. Therefore, when such mucosa specimens are obtained, this mainly concerns inflamed mucosal tissue (tonsilitis or appendicitis) and this would greatly interfere with our aim to look at the effect of the factors tested in this study".
This is an questionable statement. According to the "Kwaliteitsindicatoren 2016" of the "Inspectie voor de Gesundheitszorg" there are about 40 000 tonsillectomies and 20 000 adenectomies per year corresponding to about 25% of the yearly birth cohort. Most of these tonsillectomies are carried out after the acute inflammation has resolved. Moreover, about 16 000 apendectomies are carried out per year in the Netherlands, of which 15 - 20% of the appendices were removed although they were not inflamed. Therefore, the statement made by the authors simply seems not to be true and it would have been possible for the authors to perform their experiments with tonsillar B cells or B cells from appendices from non-inflamed tissue. Therefore, the authors should be able to include an experiment with tonsillar or appendix-derived B cells analyzing the development of interleukin-10 and IgA secreting cell.
2) Figure 1. In contrast to the statements made by the authors, purified human B cells survive perfectly well if Baff is added. The poor survival shown in figure 1 seems therefore result from poor cell culture conditions and the authors may repeat the test using synthetic media.
Supplementary Figure 1.
The authors did not perform the CD19 staining of the B cell depleted PBMC fraction. Since they use a

"negative selectio kit", the combination of antibodies in this kit does very likely not include anti-CD19 antibodies because they would have removed all B cells as well. Therefore, it would have been perfectly possible to carry out the requested staining.

Second revision – authors' response

31-Aug-2017

Response to the reviewers

First and foremost, we would like to thank the reviewers for their thorough review of the revision of our paper entitled: "BAFF augments IgA2 and IL-10 production by TLR7/8 stimulated total peripheral blood B cells".
In this rebuttal we will address the reviewers comments one by one.

Reviewer: 1

Comments to the Author
The authors followed most of the suggestions made in the first review. However, some concerns still remain. They should be resolved before publication.

Specific comments
1) In line 55pp of their response the authors state .." Although we agree with the reviewer that having mucosal B cells to compare with peripheral blood erived B cells would have been great, however, we cannot obtain these samples. In the Netherlands, it is not customary to preventively remove tonsils or appendices anymore (which was done in the past) so it is very rare to get baseline specimens. Therefore, when such mucosa specimens are obtained, this mainly concerns inflamed mucosal tissue (tonsilitis or appendicitis) and this would greatly interfere with our aim to look at the effect of the factors tested in this study".
This is an questionable statement. According to the "Kwaliteitsindicatoren 2016" of the "Inspectie voor de Gesundheitszorg" there are about 40 000 tonsillectomies and 20 000 adenectomies per year corresponding to about 25% of the yearly birth cohort. Most of these tonsillectomies are carried out after the acute inflammation has resolved. Moreover, about 16 000 apendectomies are carried out per year in the Netherlands, of which 15 - 20% of the appendices were removed although they were not

inflamed. Therefore, the statement made by the authors simply seems not to be true and it would have been possible for the authors to perform their experiments with tonsillar B cells or B cells from appendices from non-inflamed tissue. Therefore, the authors should be able to include an experiment with tonsillar or appendix-derived B cells analyzing the development of interleukin-10 and IgA secreting cell.

Unfortunately we are not in a position at this time to perform these studies in the near future.
Apart from the fact that mucosal tissues (although surgically removed in our hospitals) are difficult to obtain, we must first apply for Medical Ethical Approval, a process which can take several months. While we agree that the comparison between peripheral blood and mucosal B cells would be great, we feel that analysing mucosal B cells is a separate project altogether. In addition, it is debateable whether dissected tonsils that have been inflamed (although not anymore at the moment of dissection) are 'baseline' in terms of mucosal B cells. One would expect a higher percentage of activated and memory B cells after the inflammatory event, even if the inflammation itself is no longer ongoing. Furthermore, we clearly state in our manuscript that we use peripheral blood B cells and BAFF is also a factor found in serum. In our opinion, this makes our data in its present form relevant for readers of the European Journal of Immunology.

2) Figure 1. In contrast to the statements made by the authors, purified human B cells survive perfectly well if Baff is added. The poor survival shown in figure 1 seems therefore result from poor cell culture conditions and the authors may repeat the test using synthetic media.

We argue in our manuscript that it is not the poor cell culture conditions why B cells do not survive upon adding BAFF, it is the purity of these B cells. Most studies have much lower purity (or do not mention the % of purity). For example, in Smulski et al , Cell reports, 2017, references for isolation of B cells are given that refer to papers not giving % of purity. Here, we observed that even a small contamination of T cells or monocytes could change the viability and response of the B cells.

Supplementary Figure 1.
The authors did not perform the CD19 staining of the B cell depleted PBMC fraction. Since they use a "negative selectio kit", the combination of antibodies in this kit does very likely not include anti-CD19 antibodies because they would have removed all B cells as well. Therefore, it would have been perfectly possible to carry out the requested staining.

Our main objective was to look at pure B cells in the absence of T cells. Since we observed that adding in very low percentages already changes the viability and IgA production of these B cells, we decided to include these results in the supplementary figures. The experiments are already performed and we did

not control for contaminating B cells in this PBMC fraction, so even if we redo the staining on new experiments, still this would not prove that in our previous experiments we did not have CD19+ B cell contamination. Therefore on this point, we still stand by our earlier response namely:

'On the basis of the data however using different amounts of PBMCs depleted from B cells we feel that the contribution of contamination of B cells to the viability is unlikely. We use the B cell depleted fraction of PBMCs either 200.000 or 500.000 per well. If we would assume that the PBMC fraction has still 3% of B cells left (which probably is an overestimation, normally B cells account for 1-7% of the PMBCs, and those percentages we see as yield after negative selection), that would yield up to 6.000 B cells extra (for the 200.000) or 15.000 B cells extra (for the 500.000) on top of the 500.000 B cells used. Although we do not have formal proof, these numbers are very unlikely to account for the increase of 20% or 40 % in viability respectively.'
Reviewer: 2

Comments to the Author
The study is conducted to address the question if T-cell independent factors BAFF, APRIL, IL-10 and Retinoic acid mediates IgA production in the setting of a viral or bacterial infection. Further, the study specifically investigate immunoglobulin production in naïve and mature B cells to address which subtype that are responsible for the IgA production. Based on the results showing increased IgA2 and IL-10 production by mature B cells due to BAFF + TLR7/8 stimulation and the fact that BAFF is present in breast milk, the authors suggest that BAFF treatment might improve mucosal immunity in infants.

General
The manuscript has improved due to the addition of the new manuscripts and the rewriting. There are still some inconsistency in the language throughout the paper. For example, in several places the word "and" is used where is most likely should say "or". One example is page 14, row 99: "Although BAFF in combination with CpG-ODN and R848…" It should be "…BAFF in combination with CpG-ODN or R848…" since no experiments using CpG-ODN and R848 in combination was conducted. Please go over and change and to or where it is appropriate to reduce confusion.
We apologise for the fact that this was not changed in all instances in the last revision. We now made sure that everywhere it reads CpG-ODN or R848.

Labeling of some figures is not enough. For example, the Y-axis in most of the flow cytometry plots are just labeled with the fluorochrome used. It is better and easier to understand the graph if it is labeled with the actually marker measured. In supplemental figure 4 for example, the Y-axis here is indicating CCR10 or beta7 but instead says "APC" or "Pacific Blue" (except one of the graphs which says APC-CCR10).

Changed accordingly in Figures 1 and 2 and Supplementary figure 4

Abstract

European Journal of Immunology

The wording “viral or bacterial stimulation” should be changed to “viral or bacterial infection.” Same thing with “mimicking viral/bacterial stimulation.” TLR stimulated is correct though.
We have changed the wording and highlighted the changed text.

Results
Figure 1B and 1C: The Y-axis of the two graphs are different even though they show the same thing. In addition, to make it clearer, add “Viability of total B cells” as a header for 1B and “viability of naïve B cells” as header for 1C. This should be done for all the graphs that either look at the total B cells population or the naïve B cells.

Changed accordingly

Supplemental figure 1. In the text the authors state that they observe IL-10 receptors on both naïve and memory B cells. What is this interpretation based on when according to supplemental figure 1D there are almost no IL-10 receptor positive cells? Especially not in memory B cell population. The percentage of IL-10R in the memory population is as low as the percentage of BMCA expression, which the authors state that they “hardly observe.”
We agree with the reviewer and have adapted the text accordingly so now it states that also IL-10R was hardly observed (line 123-125).

Figure 2A-C. Even though tested, nothing is mentioned regarding the non-existing effect of APRIL or IL-10 on CD38 expression. It should at least be stated in one sentence.
We now mention this non-existing effect in lines 144-148.

Why was CD138 expression only investigated in the naive B cells population and not among total B cells? Especially when viability, and IgA and cytokine production is the highest.
Our primary read-out for plasma cell formation was IgA1 and IgA2 antibody production. For the prior revision we did only repeat the entire set of experiments on naive B cells and we therefore we able to include CD138. We did not choose to repeat the entire set of experiments on the total B cell pool only to include CD138, since our primary read-out (antibody production) already shows we have antibody secreting (plasma) cells.

Page 15, row 116 says “mucosal factor” when in the rest of the manuscript BAFF, APRIL, IL-10 and RA is no longer called mucosal factors.
We apologise that we missed this one in the last revision. We deleted the word ‘mucosal’ (also in response to reviewer 1).

Figure 5. The test regarding figure 5 is a little confusing. First the authors, correctly, state that BAFF in combination with either R848 or CpG mediate a significant increase in IL-10. Then right after, when looking at naïve B cells, the authors only mention the results for BAFF+R848 stimulation even though BAFF+CpG stimulation support the conclusion they draw (that mature B cells are responsible for the

production of IL-10).

We apologize for the confusion and have adapted our text accordingly.

Page 16, row 137: “This was also not observed….” This sentence is unclear. If the data (not shown) show that BAFF+IL10 and APRIL+IL-10 does not induce IgA production, it is clearer to write “similar results were observed with…..”
We agree with the reviewer and have adapted the text accordingly.

Material and Methods
Page 24, row 280: “ IgM and IgM in supernatant….” Should be “IgM and IgG…”
Changed accordingly

Third Editorial Decision

05-Sep-2017

Dear Dr. Brugman,

It is a pleasure to provisionally accept your manuscript entitled "BAFF augments IgA2 and IL-10 production by TLR7/8 stimulated total peripheral blood B cells." for publication in the European Journal of Immunology. For final acceptance, please follow the instructions below and return the requested items as soon as possible as we cannot process your manuscript further until all items listed below are dealt with.

Please note that EJI articles are now published online a few days after final acceptance (see Accepted Articles: http://onlinelibrary.wiley.com/journal/10.1002/(ISSN)1521-4141/accepted). The files used for the Accepted Articles are the final files and information supplied by you in Manuscript Central. You should therefore check that all the information (including author names) is correct as changes will NOT be permitted until the proofs stage.

We look forward to hearing from you and thank you for submitting your manuscript to the European Journal of Immunology.

Yours sincerely,

Eloho Etemire

on behalf of

Prof. Andreas Radbruch

Dr. Eloho Etemire

Editorial Office

European Journal of Immunology

e-mail: ejied@wiley.com

www.eji-journal.eu
